# Topology independent protein structural alignment

**DOI:** 10.1186/1471-2105-8-388

**Published:** 2007-10-15

**Authors:** Joe Dundas, TA Binkowski, Bhaskar DasGupta, Jie Liang

**Affiliations:** 1 Department of Bioengineering, University of Illinois at Chicago, Chicago, IL 60607-7052, USA; 2 Department of Computer Science, University of Illinois at Chicago, Chicago, IL 60607-7053, USA

## Abstract

**Background:**

Identifying structurally similar proteins with different chain topologies can aid studies in homology modeling, protein folding, protein design, and protein evolution. These include circular permuted protein structures, and the more general cases of non-cyclic permutations between similar structures, which are related by non-topological rearrangement beyond circular permutation. We present a method based on an approximation algorithm that finds sequence-order independent structural alignments that are close to optimal. We formulate the structural alignment problem as a special case of the maximum-weight independent set problem, and solve this computationally intensive problem approximately by iteratively solving relaxations of a corresponding integer programming problem. The resulting structural alignment is sequence order independent. Our method is also insensitive to insertions, deletions, and gaps.

**Results:**

Using a novel similarity score and a statistical model for significance *p*-value, we are able to discover previously unknown circular permuted proteins between nucleoplasmin-core protein and auxin binding protein, between aspartate rasemase and 3-dehydrogenate dehydralase, as well as between migration inhibition factor and arginine repressor which involves an additional strand-swapping. We also report the finding of non-cyclic permuted protein structures existing in nature between AML1/core binding factor and ribofalvin synthase. Our method can be used for large scale alignment of protein structures regardless of the topology.

**Conclusion:**

The approximation algorithm introduced in this work can find good solutions for the problem of protein structure alignment. Furthermore, this algorithm can detect topological differences between two spatially similar protein structures. The alignment between MIF and the arginine repressor demonstrates our algorithm's ability to detect structural similarities even when spatial rearrangement of structural units has occurred. The effectiveness of our method is also demonstrated by the discovery of previously unknown circular permutations. In addition, we report in this study the finding of a naturally occurring non-cyclic permuted protein between AML1/Core Binding Factor chain F and riboflavin synthase chain A.

## Background

The classification of protein structures often rely on the topology of secondary structural elements. For example, the Structural Classification of Proteins (SCOP) system classifies proteins structures into common folds using the topological arrangement of secondary structural units [1]. Most protein structural alignment methods can reliably classify proteins into similar folds given the structural units from each protein are in the same sequential order. However, the evolutionary possibility of proteins with different structural topology but with similar spatial arrangement of their secondary structures pose a problem. One such possibility is the circular permutation.

**Figure 1:**
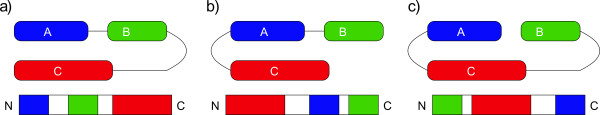
**Circular permutation example**. The cartoon illustration of three protein structures whose domains are similarly arranged in space but appear in different order in primary sequences. The location of domains A, B, C in primary sequences are shown in a layout below each structure. Their orderings are related by circular permutation [2].

A circular permutation is an evolutionary event that results in the N and C terminus transferring to a different position on a protein. Figure 1[2] shows a simplified example of circular permutation. There are three proteins, all consist of three domains (A, B, and C). Although the spatial arrangement of the three domains are very similar, the ordering of the domains in the primary sequence has been circularly permuted. Lindqvist *et. al*. observed the first natural occurrence of a circular permutation between jackbean concanavalin A and favin [3]. Although the jackbean-favin permutation was the result of post-translational ligation of the N and C terminus and cleavage elsewhere in the chain, a circular permutation can arise from events at the gene level through gene duplication and exon shuffling.

Permutation by duplication [4,5] is a widely accepted model where a gene first duplicates and fuses. After fusion, a new start codon is inserted into one gene copy while a new stop codon is inserted into the second copy. Peisajovich *et al*. demonstrated the evolutionary feasibility of permutation via duplication by creating functional intermediates at each step of the *permutation by duplication model *for DNA methyltransferases [6]. Identifying structurally similar proteins with different chain topologies, including circular permutation, can aid studies in homology modeling, protein folding, and protein design. An algorithm that can structurally align two proteins independent of their backbone topologies would be an important tool.

The biological implications of thermodynamically stable and biologically functional circular permutations, both natural and artificial, has resulted in much interest in detecting circular permutations in proteins [3,7-11]. The more general problem of detecting non-topological structural similarities beyond circular permutation has received less attention. We refer to these as *non-cyclic permutations *from now on. Tabtiang *et al*. were able to create a thermodynamically stable and biologically functional non-cyclic permutation, indicating that non-cyclic permutations may be as important as circular permutations [12]. In this study, we present a novel method that detects spatially similar structures that can identify structures related by circular and more complex non-cyclic permutations. Detection of non-cyclic permutation is possible by our algorithm by virtue of a recursive combination of a local-ratio approach with a global linear-programming formulation. This paper is organized as follows. We first show that our algorithm is capable of finding known circular permutations with sensitivity and specificity. We then report the discovery of three new circular permutations and one example of a non-cyclic permutation that to our knowledge have not been reported in literature. We conclude with remarks and discussions.

## Results and discussion

For availability of our alignment software please see [13].

### Detection of known circular permutations

We first demonstrate the ability of our algorithm to detect circular permutations by examining known examples of circular permutations. The results are summarized in Table 1 and Table 2.

**Table 1:** Known circular permutation results

Protein 1	Protein 2	Us			DaliLite		K2	
PDB(Length)	PDB(Length)	*N*	RMSD	*p *– *value*	*N*	RMSD	*N*	RMSD

1rinA(180)	2cna_(237)	152*	**0.875**	10^-6^	106	1.7	60	0.92
1glh_(214)	1cpn_(208)	192*	**1.163**	10^-5^	156	0.4	156	0.41
1exg_(110)	1tul_(102)	74*	**1.485**	10^-4^	63	4.0	34	2.26
1rhgA(145)	1bcfA(158)	118*	**1.500**	10^-4^	94	2.3	81	1.51
1ihwA(52)	1sso_(62)	46*	**0.502**	10^-3^	45	2.9	28	1.93

Comparison of results against DaliLite and K2. DaliLite is not expected to find sequence order independent alignments. K2 did not find the circular permutation even when the sequence order independent options was selected.

**Table 2:** Known circular permutation results

Protein 1	Protein 2	Us			MASS		OPAAS		SAMO		Topofit	
PDB(Length)	PDB(Length)	*N*	*R*	*p*	*N*	*R*	*N*	*R*	*N*	*R*	*N*	*R*

1rinA(180)	2cna_(237)	152*	**0.875**	10^-6^	164*	1.2	167*	1.48	174*	1.581	152*	1.09
1glh_(214)	1cpn_(208)	192*	**1.163**	10^-5^	206*	0.49	No	solution	170*	3.283	206*	0.49
1exg_(110)	1tul_(102)	74*	**1.485**	10^-4^	60*	1.9	No	solution	93*	2.88	52*	1.79
1rhgA(145)	1bcfA(158)	118*	**1.500**	10^-4^	106*	1.7	63*	2.12	126*	2.309	109*	1.4
1ihwA(52)	1sso_(62)	46*	**0.502**	10^-3^	39*	1.7	No	solution	48*	2.713	35*	1.47

Comparison of our alignment results with that of MASS, OPAAS, SAMO, and Topofit for known circular permutations. Each method detected the circular permutations. Our method normally returns more equivalent residues at a lower RMSD. *N *indicates the number of aligned residues. An * next to the number of aligned residues indicates that a circular permutation was found. *R *indicates the cRMSD of the alignment. *p *indicates the *p*-value of our alignment.

In Table 1 we compare results against DaliLite and K2. As expected, DaliLite returned the largest sequential alignment. K2 did not find circular permutations even when the option to ignore sequence order constraints was selected.

In Table 2 we compare our alignment results to the methods of MASS [14], OPAAS [15], SAMO [7], and Topofit [16]. Each method is able to detect circular permutations. However, Table 2 shows that our method normally finds more equivalent residues with a lower RMSD. Compared with SAMO our method found less aligned residues in 4 of the 5 shown alignments. However, our *cRMSD *values are considerably better. At the time of this writing, SAMO only outputs the *cRMSD *and the number of equivalent residues (N) of the alignment, without specifying the residue equivalence relationships between the two aligned protein structures. This makes it difficult to compare the quality of the alignments. Table 2 shows that our method finds better alignments in terms of cRMSD than other structural alignment methods when the two proteins are related by a circular permutation.

The GANSTA method by Kolbeck et al [17] can also align similar structures independent of the connectivity. The approach is somewhat similar to the Blast method in sequence alignment, where a set of seeds of high-similarity pairs of secondary structural elements (SSE) are first identified, and are then aligned through a genetic algorithm, regardless of the connectivity.

The SCALI method by Yuan and Bystroff [18] assembles from a library of gapless alignment of fragments of local sequence-structure hierarchically, enforcing compactness and conserved contacts, but disregard the sequence ordering of the fragments. The aligned local fragments are then incremented by adding a new fragment pair. This process is organized as a tree, where nodes corresponds to the addition of new fragments. A breadth-first tree search method was then carried out, with a number of heuristic conditions to limit the search space.

Instead of only aligning regular SSE fragments, our method differs from GANSTA and has no restriction on spatial patterns belonging to a regular SSE, and therefore is also applicable to loop regions. Our method differs from SCALI in that our fragments are not prebuilt, but are exhaustive fragments ranging from size 4–7. Compared to both methods, our method provides a guaranteed optimal ratio of aligned structures, whereas the heuristics employed by GANSTA and SCALI cannot guarantee that a good alignment can be found, and when an alignment is found, there is no guarantee that it will be within a certain ratio of the best possible alignment. In practice, we find that GANSTA often requires 3–5 hours for aligning a pair of proteins, and sometimes no results are returned. In contrast, our method usually terminates between 30 seconds – 5 minutes. The SCALI website consists of pre-computed results of aligned structures and does not allow user input for a customized alignment, therefore it is difficult to compare performance of our method with SCALI on the examples reported in Table 2.

### Discovery of novel circular permutations and a novel non-cyclic permutation

The effectiveness of our method is also demonstrated by the discovery of previously unknown circular permutations. In an attempt to test our algorithm's ability to discover new circular permutations, we structurally aligned a subset of 3,336 structures from PDBSELECT 90% [19]. We first selected proteins from PDBSELECT90 (sequences have less than 90% identities) whose N and C termini were no further than 30Å apart. From this subset of 3,336 proteins, we aligned two proteins if they met the following conditions: the difference in their lengths was no more than 75 residues, and they had approximately the same secondary structure content. To compare secondary structure content, we determined the percentage of the residues labeled as helix, strand, and other for each structure. Two structures were considered to have the same secondary structure content if the difference between each secondary structure label was less than 10%. Within the approximately 200,000 alignments, we found 426 candidate circular permutations. Of these circular permutations, 312 were symmetric proteins that can be aligned with or without a circular permutation. Of the 114 non-symmetric circular permutations, 112 were already known in literature, and 3 are novel. We describe these three novel circular permutations as well as a novel non-cyclic permutation in some details.

### Nucleoplasmin-core and auxin binding protein

**Figure 2:**
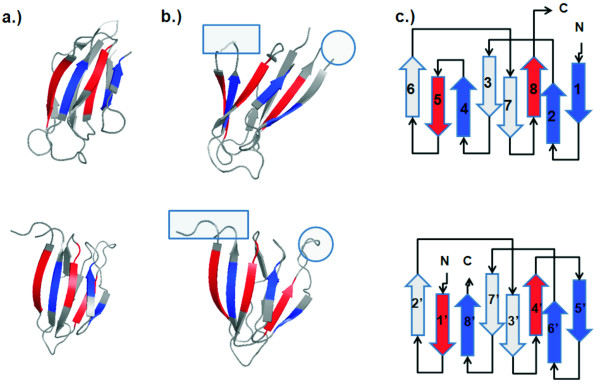
**Nucleoplasmin-core and auxin binding protein 1**. A new circular permutation discovered between nucleoplasmin-core (1k5j, chain E, top panel), and the fragment of residues 37–127 of auxin binding protein 1 (1lrh, chain A, bottom panel). a) These two proteins superimpose well spatially, with an RMSD value of 1.36Å for an alignment length of 68 residues and a significant *p*-value of 2.7 × 10^-5 ^after Bonferroni correction. b) These proteins are related by a circular permutation. The short loop connecting strand 4 and strand 5 of nucleoplasmin-core (in rectangle, top) becomes disconnected in auxin binding protein 1. The N- and C- termini of nucleoplasmin-core (in ellipse, top) become connected in auxin binding protein 1 (in ellipse, bottom). For visualization, residues in the N-to-C direction before the cut in the nucleoplasmin-core protein are colored red, and residues after the cut are colored blue. c) The topology diagram of these two proteins. In the original structure of nucleoplasmin-core, the electron density of the loop connecting strand 4 and strand 5 is missing.

The first novel circular permutation we found was between the nucleoplasmin-core protein in *Xenopu laevis *(PDB ID 1k5j, chain E) [20] and the auxin binding protein in maize (PDB ID 1lrh, chain A, residues 37 through 127) [21]. The overall structural alignment between 1k5jE (Figure 2a, top) and 1lrhA (Figure 2a, bottom) has an RMSD value of 1.36Å with an alignment length of 68 residues and a significant *p*-value of 2.7 × 10^-5 ^after Bonferroni correction. These proteins are related by a circular permutation. The short loop connecting two antiparallel strands in nucleoplasmin-core protein (in ellipse, top of Fig 2b) becomes disconnected in auxin binding protein 1 (in ellipse, bottom of Fig 2b), and the N- and C- termini of the nucleoplasmin-core protein (in square, top of Fig 2b) are connected in auxin binding protein 1 (square, bottom of Fig 2b).

### Aspartate racemase and type II 3-dehydrogenate dehyrdalase

**Figure 3:**
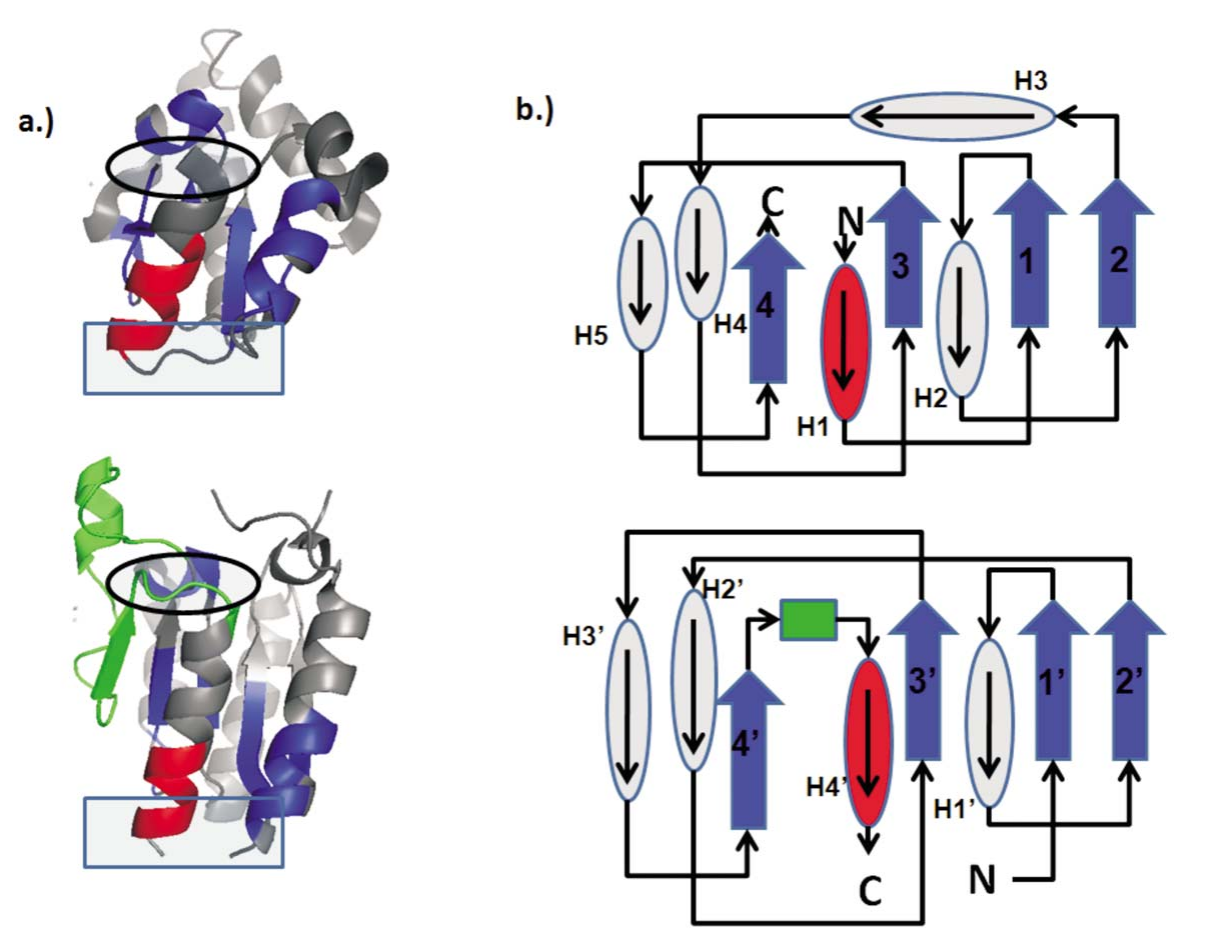
**Aspartate racemase and type II 3-deydrogenate dehyralase**. A new circular permutation discovered between a) aspartate racemase (1iu9, chain A, top) and type II 3-dehydrogenate dehydralase (1h0r, chain A, bottom) superimpose well spatially with an RMSD of 1.49Å between 59 residues, with a significant *p*-value of 4.7 × 10^-4^. b) These proteins are related by a circular permutation. The loop connecting helix 1 with strand 1 in aspartate racemase (in rectangle, top) becomes disconnected in type II 3-dehydrogenate dehydralase (in rectangle, bottom), but the N- and C- termini of aspartate racemase (in ellipse, top) becomes connected in dehydrogenate dehydralase (in ellipse, bottom) with an insertion (shown in green). For visualization, residues of aspartate racemase in the N-to-C direction before the cut in the dehydrogenate dehydralase are colored red, and residues after the cut are colored blue. c) The topology diagram of these two proteins. Here an ellipse represents a helix and a block arrow represents a strand.

Another circular permutation we found is between the aspartate racemase (PDB ID 1iu9, chain A) [22] and type II 3-dehydrogenate dehydralase (PDB ID 1h0r, chain A) [23]. The overall structural alignment between 1iu9A (Figure 3a, top) and 1h0rA (Figure 3a, bottom) has an RMSD value of 1.49Å with an alignment length of 59 residues and a significant *p*-value of 4.7 × 10^-4 ^after Bonferroni correction. These proteins are related by a circular permutation. The loop connecting the first helix with the first strand in aspartate racemase (in rectangle, top of Figure 3b) becomes disconnected in 3-dehydrogenate dehydralase (in rectangle, bottom), while the N- and C-termini of the aspartate racemase (in ellipse, top) are connected in the dehydralase by an insertion (shown in green) (Figure 3b, bottom). Figure 3c depicts the topology of these two proteins.

### Migration inhibition factor and arginine repressor

**Figure 4:**
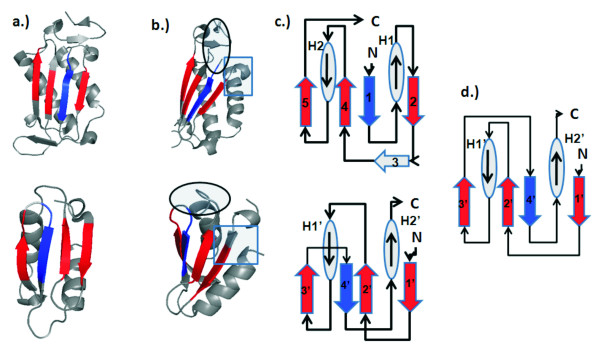
**Microphage migration inhibition factor and C-terminal domain of arginine repressor**. A new circular permutation discovered between a) the microphage migration inhibition factor (MIF, PDB ID 1uiz, chain A, top) and the C-terminal domain of arginine repressor (AR, 1xxa, chain C, bottom). a) These two proteins superimpose well spatially, with a RMSD of 1.74Å for an alignment length of 24 residues, and a *p*-value of 1.3 × 10^-2^. b.) These proteins are related by a circular permutation. The loop connecting helix 1 with strand 2 of MIF (in rectangle, top) becomes disconnected in arginine repressor, the N- and C- termini of MIF (in ellipse, top) becomes connected in arginine repressor (in ellipse, bottom). The disconnection of helix 1 from strand 2 of MIF removes some spatial constraints, allowing strand 1' in AR to swap places with strand 4'. c) The topology diagram of these two proteins. d.) The artificial topology diagram for arginine repressor, where strand 2' and strand 4' are spatially swapped back. The diagram for AR in (c) has the same topology as the diagram in (d).

The majority of circular permutations maintain their overall three dimensional structures. However, it is possible that additional structural changes may occur beyond circular permutation. We have discovered a novel circular permutation between the microphage migration inhibition factor (MIF, PDB ID 1uiz, chain A, from *Xenopus laevis*) and the C-terminal domain of arginine repressor (AR, 1xxa, chain C, from *Escherichia coli*) [24,25], which contains in addition to circular permutation a spatial swapping of two antiparallel strands, and a change in the orientation of a helix. The overall folds of these two protein are different by the SCOP definition. The MIF factor belongs to the tautomerase fold, and the C-terminal domain of arginine repressor belongs to the DCoH-like fold. The overall structural alignment between 1uiz chain A (Figure 4a, top) and 1xxa chain C (Figure 4a, bottom) has an RMSD value of 1.74Å between 24 residues, with a *p*-value of 1.3 × 10^-2 ^after Bonferroni correction. They are related by a circular permutation. The short loop of MIF (Figure 4b, top, in rectangle) connecting the first helix and the second strand from the N-terminus becomes disconnected in arginine repressor (AR, Figure 4a, bottom, in rectangle). The relaxing of spatial constraints imposed by the connection allows strand 1 of MIF to swap positions with strand 4 of MIF. This can also be clearly seen in Figure 4a, where a strand colored in red (strand 2' in AR, corresponding to strand 4 in MIF) swaps position with the strand colored in blue (strand 4' in AR, corresponding to strand 1 in MIF). Although the strands have changed positions spatially, their topology remains the same (Figure 4c and 4d). The circular permutation and strand swapping cause additional structural changes. In MIF, helix 1 was connected with a short loop to strand 2 (Figure 4b, top, in rectangle). With the creation of the new N- and C-termini replacing the original short loop (Figure 4b, bottom, rectangle), helix 1 loses the spatial constraints imposed by the connection, and was pulled over when strand 1 and strand 4 swap positions. The net results for helix 1 is that its orientation in arginine repressor (Figure 4b, bottom) is almost perpendicular to its original orientation in MIF (Figure 4b, top).

### Beyond circular permutation

**Figure 5:**
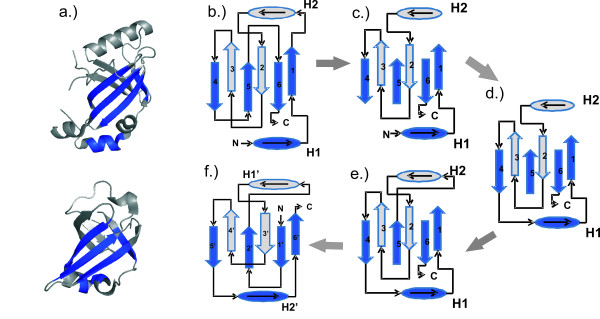
**A non-cyclic permutation**. A novel non-cyclic permutation discovered between AML1/Core Binding Factor (AML1/CBF, PDB ID 1e50, Chain F, top) and riboflavin synthase (PDBID 1pkv, chain A, bottom) a) These two proteins superimpose well spatially, with an RMSD of 1.23 Å and an alignment length of 42 residues, with a significant *p*-value of 2.8 × 10^-4 ^after Bonferroni correction. Aligned residues are colored blue. b) These proteins are related by multiple permutations. The steps to transform the topology of AML1/CBF (top) to riboflavin (bottom) are as follows: c) Remove the the loops connecting strand 1 to helix 2, strand 4 to strand 5, and strand 5 to helix 6; d) Connect the C-terminal end of strand 4 to the original N-termini; e) Connect the C-terminal end of strand 5 to the N-terminal end of helix 2; f) Connect the original C-termini to the N-terminal end of strand 5. The N-terminal end of strand 6 becomes the new N-termini and the C-terminal end of strand 1 becomes the new C-termini. We now have the topology diagram of riboflavin synthase.

The information that naturally occurring circular permutations contain about the folding mechanism of proteins has led to a lot of interest in their detection. Another interesting class of permuted proteins is the non-cyclic permutation. Although there has been previous work on the detection of non-cyclic permutations [14-16,26], compared to cyclic-permutations there has been relatively little research of noncyclic-permutations. As an example of this important class of topologically permuted proteins, Tabtiang *et al *(2004) were able to artificially create a noncyclic permutation of the Arc repressor that was thermodynamically stable, refolds on the sub-millisecond time scale, and binds operator DNA with nanomolar affinity [12]. This raises the question of whether or not these non-cyclic permutations can arise naturally. Here we report the discovery of a *possibly *naturally occurring non-cyclic permutation between chain F of AML1/Core Binding Factor (AML1/CBF, PDB ID 1e50, Figure 5, top) and chain A of riboflavin synthase (PDB ID 1pkv, Figure 5a, bottom) [27,28]. The two structures align well with a RMSD of 1.23 Å with an alignment length of 42 residues, and a significant *p*-value of 2.8 × 10^-4 ^after Bonferroni correction. The topology diagram of AML1/CBF (Figure 5b) can be transformed into the topology diagram of riboflavin synthase (Figure 5f) by the following steps: Remove the the loops connecting strand 1 to helix 2, strand 4 to strand 5, and strand 5 to strand 6 (Figure 5c). Connect the C-terminal end of strand 4 to the original N-termini (Figure 5d). Connect the C-terminal end of strand 5 to the N-terminal end of helix 2 (Figure 5e). Connect the original C-termini to the N-terminal end of strand 5. The N-terminal end of strand 6 becomes the new N-termini and the C-terminal end of strand 1 becomes the new C-termini (Figure 5f).

### Algorithm comparison

Zhu *et al *(2005) demonstrated the quality of their structural alignment algorithm (FAST [29]) by comparing their alignments with manually curated alignments in the HOMSTRAD database [30]. As of March 2007, HOMSTRAD contains 3,454 proteins structures in 1,032 families. We randomly chose 10 structures from families that consisted of more than 20 protein structures. Within each family, we compared the structures using our alignment method to determine accuracy. Within alignments, our method's predicted equivalent residues agreed with HOMSTRAD 93% of the time. Discrepancies occur normally when our method would shift a fragment pair by one or two residues along the backbone. Zhu *et al*. chose 11 representatives from different structural classes as examples (Table IV of [29]). Table 3 is a representation of Table IV from [29] comparing our results with that of FAST [29] and DaliLite [31]. In all alignments, our method found sequentially ordered alignments, therefore, there is no bias in favor of our sequence order independent method. It can be seen from Table 3 that the equivalent residues that our method predicts are consistent with the manually determined residues of HOMSTRAD.

**Table 3:** Alignment quality

Proteins		HOMSTRAD		FAST			US		
PDB(PDB)	PDB(PDB)	N	RMSD	N	M%	RMSD	N	M%	RMSD

1dfaA	1qceA	57	2.5	55	55%	1.2	**45**	**72%**	**1.1**
1hx8A	1hg5A	258	1.1	255	99%	1.1	**247**	**98%**	**1.0**
2ahjA	1rieA	192	4.3	187	89%	2.0	**168**	**99%**	**1.3**
1h7sA	1b63A	105	2.2	98	99%	2.0	**96**	**100%**	**1.9**
1ed9A	1ew2A	403	5.6	343	98%	1.7	**252**	**100%**	**1.2**
1oyc_	2tmdA	330	3.6	284	97%	2.3	**193**	**94%**	**1.4**
1fjnA	1ica_	33	4.7	28	100%	1.9	**33**	**100%**	**1.8**
1tpn_	1fbr_	43	2.4	40	93%	2.2	**39**	**97%**	**2.2**
1e12A	1c3wA	220	1.7	214	97%	1.5	**170**	**100%**	**0.9**
1af6A	1a0tP	377	4.6	323	97%	1.8	**281**	**97%**	**1.5**
1hc1_	1lla_	582	2.3	546	97%	1.7	**380**	**100%**	**1.4**

Table IV from Zhu *et al*. (2005) with the addition of our alignment results. Zhu *et al*. chose the following alignment examples to cover a broad range of structural classes. For each alignment, our method returned sequence ordered alignments. N is the number of aligned residues corresponding to each method and M% is the number of aligned residues generated by the corresponding algorithm that are equivalent to HOMSTRAD's aligned residues.

## Conclusion

The approximation algorithm introduced in this work can find good solutions for the problem of protein structure alignment. Furthermore, this algorithm can detect topological differences between two spatially similar protein structures. The alignment between MIF and the arginine repressor demonstrates our algorithm's ability to detect structural similarities even when spatial rearrangement of structural units has occurred. In addition, we report in this study the finding of a naturally occurring non-cyclic permuted protein between AML1/Core Binding Factor chain F and riboflavin synthase chain A.

In our method, the scoring function plays a pivotal role in detecting substructure similarity of proteins. We expect future experimentation on optimizing the parameters used in our similarity scoring system can improve detection of topologically independent structural alignment. In this study, we were able to fit our scoring system to an Extreme Value Distribution (EVD), which allowed us to perform an automated search for circular permuted proteins. Although the *p*-value obtained from our EVD fit is sufficient for determining the biological significance of a structural alignment, the structural change between the microphage migration inhibition factor and the C-terminal domain of arginine repressor (Figure 3) indicates a need for a similarity score that does not bias heavily towards cRMSD measure for scoring circular permutations.

Whether naturally occurring circular permutations are frequent events in the evolution of protein genes is currently an open question. Lindqvist *et al*. (1997) pointed out that when the primary sequences have diverged beyond recognition, circular permutations may still be found using structural methods [3]. In this study, we discovered three examples of novel circularly permuted protein structures and a non-cyclic permutation among 200,000 protein structural alignments for a set of non-redundant 3,336 proteins. This is an incomplete study, as we restricted our studies to proteins whose N- and C- termini distance were less than 30Å. We plan to relax the N to C distance and include more proteins in future work to expand the scope of the investigation.

## Methods

### Approach

In this study, we describe a new algorithm that can align two protein structures or substructures independent of the connectivity of their secondary structure elements. We first exhaustively fragment the two proteins separately. An approximation algorithm based on a fractional version of the local-ratio approach for scheduling split-interval graphs [32] is then used to search for the combination of peptide fragments from both structures that will optimize the global alignment of the two structures.

**Figure 6:**
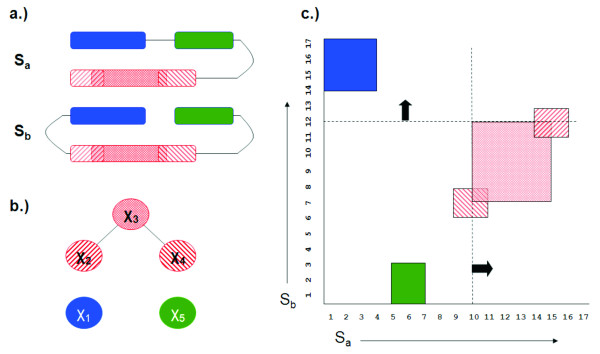
**Implementation example with vertex sweep**. An illustration of the first iteration of our algorithmic approaches for *BSSI*_Λ, *σ*_: a) The cartoon representation of circularly permuted proteins *S*_*a *_and *S*_*b*_; b) The problem represented as a graph where each node *χ*_*i *_∈ Λ represents an aligned fragment pair and each edge represents two inconsistent pairs; c) An illustration how sweep lines (dashed) can identify inconsistent aligned pairs as required to generate the interval clique inequalities. A rectangle is an ordered fragment pair (e.g., the solid green rectangle is the pair *χ*_5 _= (λ5,3a,λ1,3b
 MathType@MTEF@5@5@+=feaafiart1ev1aaatCvAUfKttLearuWrP9MDH5MBPbIqV92AaeXatLxBI9gBaebbnrfifHhDYfgasaacH8akY=wiFfYdH8Gipec8Eeeu0xXdbba9frFj0=OqFfea0dXdd9vqai=hGuQ8kuc9pgc9s8qqaq=dirpe0xb9q8qiLsFr0=vr0=vr0dc8meaabaqaciaacaGaaeqabaqabeGadaaakeaaiiGacqWF7oaBdaqhaaWcbaGaeGynauJaeiilaWIaeG4mamdabaGaemyyaegaaOGaeiilaWIae83UdW2aa0baaSqaaiabigdaXiabcYcaSiabiodaZaqaaiabdkgaIbaaaaa@3982@)).

The methods discussed here do resemble the methods in our previous conference paper [2]. However, they are similar because they both use the same approximation algorithm used for scheduling split interval graphs that appears in [32]. Beyond the approximation algorithm for scheduling split-interval graphs, the methods are different. Figure 1 does appear in our previous conference paper [2]. However, Figure 6 and Table 4 are different due to errors in the corresponding figure and table in that previous paper. Also, note that the previous conference version [2] had a recursive formulation of the algorithm as opposed to the *non-recursive *formulation as described in this paper. There are other differences too, including significant improvements/corrections of notations.

**Table 4:** Constraints

Interval clique inequalities:	**(2)**
yχ5,λa≤1 MathType@MTEF@5@5@+=feaafiart1ev1aaatCvAUfKttLearuWrP9MDH5MBPbIqV92AaeXatLxBI9gBaebbnrfifHhDYfgasaacH8akY=wiFfYdH8Gipec8Eeeu0xXdbba9frFj0=OqFfea0dXdd9vqai=hGuQ8kuc9pgc9s8qqaq=dirpe0xb9q8qiLsFr0=vr0=vr0dc8meaabaqaciaacaGaaeqabaqabeGadaaakeaacqWG5bqEdaWgaaWcbaacciGae83Xdm2aaSbaaWqaaiabiwda1iabcYcaSiab=T7aSnaaBaaabaGaemyyaegabeaaaeqaaaWcbeaakiabgsMiJkabigdaXaaa@37EB@	Line sweep at *a*_*t *_= 1
yχ1,λa≤1 MathType@MTEF@5@5@+=feaafiart1ev1aaatCvAUfKttLearuWrP9MDH5MBPbIqV92AaeXatLxBI9gBaebbnrfifHhDYfgasaacH8akY=wiFfYdH8Gipec8Eeeu0xXdbba9frFj0=OqFfea0dXdd9vqai=hGuQ8kuc9pgc9s8qqaq=dirpe0xb9q8qiLsFr0=vr0=vr0dc8meaabaqaciaacaGaaeqabaqabeGadaaakeaacqWG5bqEdaWgaaWcbaacciGae83Xdm2aaSbaaWqaaiabigdaXiabcYcaSiab=T7aSnaaBaaabaGaemyyaegabeaaaeqaaaWcbeaakiabgsMiJkabigdaXaaa@37E3@	Line sweep at *a*_*t *_= 5
yχ4,λa≤1 MathType@MTEF@5@5@+=feaafiart1ev1aaatCvAUfKttLearuWrP9MDH5MBPbIqV92AaeXatLxBI9gBaebbnrfifHhDYfgasaacH8akY=wiFfYdH8Gipec8Eeeu0xXdbba9frFj0=OqFfea0dXdd9vqai=hGuQ8kuc9pgc9s8qqaq=dirpe0xb9q8qiLsFr0=vr0=vr0dc8meaabaqaciaacaGaaeqabaqabeGadaaakeaacqWG5bqEdaWgaaWcbaacciGae83Xdm2aaSbaaWqaaiabisda0iabcYcaSiab=T7aSnaaBaaabaGaemyyaegabeaaaeqaaaWcbeaakiabgsMiJkabigdaXaaa@37E9@	Line sweep at *a*_*t *_= 9
yχ3,λa+yχ4,λa≤1 MathType@MTEF@5@5@+=feaafiart1ev1aaatCvAUfKttLearuWrP9MDH5MBPbIqV92AaeXatLxBI9gBaebbnrfifHhDYfgasaacH8akY=wiFfYdH8Gipec8Eeeu0xXdbba9frFj0=OqFfea0dXdd9vqai=hGuQ8kuc9pgc9s8qqaq=dirpe0xb9q8qiLsFr0=vr0=vr0dc8meaabaqaciaacaGaaeqabaqabeGadaaakeaacqWG5bqEdaWgaaWcbaacciGae83Xdm2aaSbaaWqaaiabiodaZiabcYcaSiab=T7aSnaaBaaabaGaemyyaegabeaaaeqaaaWcbeaakiabgUcaRiabdMha5naaBaaaleaacqWFhpWydaWgaaadbaGaeGinaqJaeiilaWIae83UdW2aaSbaaeaacqWGHbqyaeqaaaqabaaaleqaaOGaeyizImQaeGymaedaaa@4155@	Line sweep at *a*_*t *_= 10
yχ3,λa≤1 MathType@MTEF@5@5@+=feaafiart1ev1aaatCvAUfKttLearuWrP9MDH5MBPbIqV92AaeXatLxBI9gBaebbnrfifHhDYfgasaacH8akY=wiFfYdH8Gipec8Eeeu0xXdbba9frFj0=OqFfea0dXdd9vqai=hGuQ8kuc9pgc9s8qqaq=dirpe0xb9q8qiLsFr0=vr0=vr0dc8meaabaqaciaacaGaaeqabaqabeGadaaakeaacqWG5bqEdaWgaaWcbaacciGae83Xdm2aaSbaaWqaaiabiodaZiabcYcaSiab=T7aSnaaBaaabaGaemyyaegabeaaaeqaaaWcbeaakiabgsMiJkabigdaXaaa@37E7@	Line sweep at *a*_*t *_= 12
yχ3,λa+yχ2,λa≤1 MathType@MTEF@5@5@+=feaafiart1ev1aaatCvAUfKttLearuWrP9MDH5MBPbIqV92AaeXatLxBI9gBaebbnrfifHhDYfgasaacH8akY=wiFfYdH8Gipec8Eeeu0xXdbba9frFj0=OqFfea0dXdd9vqai=hGuQ8kuc9pgc9s8qqaq=dirpe0xb9q8qiLsFr0=vr0=vr0dc8meaabaqaciaacaGaaeqabaqabeGadaaakeaacqWG5bqEdaWgaaWcbaacciGae83Xdm2aaSbaaWqaaiabiodaZiabcYcaSiab=T7aSnaaBaaabaGaemyyaegabeaaaeqaaaWcbeaakiabgUcaRiabdMha5naaBaaaleaacqWFhpWydaWgaaadbaGaeGOmaiJaeiilaWIae83UdW2aaSbaaeaacqWGHbqyaeqaaaqabaaaleqaaOGaeyizImQaeGymaedaaa@4151@	Line sweep at *a*_*t *_= 14
yχ2,λa≤1 MathType@MTEF@5@5@+=feaafiart1ev1aaatCvAUfKttLearuWrP9MDH5MBPbIqV92AaeXatLxBI9gBaebbnrfifHhDYfgasaacH8akY=wiFfYdH8Gipec8Eeeu0xXdbba9frFj0=OqFfea0dXdd9vqai=hGuQ8kuc9pgc9s8qqaq=dirpe0xb9q8qiLsFr0=vr0=vr0dc8meaabaqaciaacaGaaeqabaqabeGadaaakeaacqWG5bqEdaWgaaWcbaacciGae83Xdm2aaSbaaWqaaiabikdaYiabcYcaSiab=T7aSnaaBaaabaGaemyyaegabeaaaeqaaaWcbeaakiabgsMiJkabigdaXaaa@37E5@	Line sweep at *a*_*t *_= 16
Interval clique inequalities:	**(3)**
yχ1,λb≤1 MathType@MTEF@5@5@+=feaafiart1ev1aaatCvAUfKttLearuWrP9MDH5MBPbIqV92AaeXatLxBI9gBaebbnrfifHhDYfgasaacH8akY=wiFfYdH8Gipec8Eeeu0xXdbba9frFj0=OqFfea0dXdd9vqai=hGuQ8kuc9pgc9s8qqaq=dirpe0xb9q8qiLsFr0=vr0=vr0dc8meaabaqaciaacaGaaeqabaqabeGadaaakeaacqWG5bqEdaWgaaWcbaacciGae83Xdm2aaSbaaWqaaiabigdaXiabcYcaSiab=T7aSnaaBaaabaGaemOyaigabeaaaeqaaaWcbeaakiabgsMiJkabigdaXaaa@37E5@	Line sweep at *b*_*t *_= 1
yχ4,λb≤1 MathType@MTEF@5@5@+=feaafiart1ev1aaatCvAUfKttLearuWrP9MDH5MBPbIqV92AaeXatLxBI9gBaebbnrfifHhDYfgasaacH8akY=wiFfYdH8Gipec8Eeeu0xXdbba9frFj0=OqFfea0dXdd9vqai=hGuQ8kuc9pgc9s8qqaq=dirpe0xb9q8qiLsFr0=vr0=vr0dc8meaabaqaciaacaGaaeqabaqabeGadaaakeaacqWG5bqEdaWgaaWcbaacciGae83Xdm2aaSbaaWqaaiabisda0iabcYcaSiab=T7aSnaaBaaabaGaemOyaigabeaaaeqaaaWcbeaakiabgsMiJkabigdaXaaa@37EB@	Line sweep at *b*_*t *_= 6
yχ4,λb+yχ3,λb≤1 MathType@MTEF@5@5@+=feaafiart1ev1aaatCvAUfKttLearuWrP9MDH5MBPbIqV92AaeXatLxBI9gBaebbnrfifHhDYfgasaacH8akY=wiFfYdH8Gipec8Eeeu0xXdbba9frFj0=OqFfea0dXdd9vqai=hGuQ8kuc9pgc9s8qqaq=dirpe0xb9q8qiLsFr0=vr0=vr0dc8meaabaqaciaacaGaaeqabaqabeGadaaakeaacqWG5bqEdaWgaaWcbaacciGae83Xdm2aaSbaaWqaaiabisda0iabcYcaSiab=T7aSnaaBaaabaGaemOyaigabeaaaeqaaaWcbeaakiabgUcaRiabdMha5naaBaaaleaacqWFhpWydaWgaaadbaGaeG4mamJaeiilaWIae83UdW2aaSbaaeaacqWGIbGyaeqaaaqabaaaleqaaOGaeyizImQaeGymaedaaa@4159@	Line sweep at *b*_*t *_= 7
yχ3,λb≤1 MathType@MTEF@5@5@+=feaafiart1ev1aaatCvAUfKttLearuWrP9MDH5MBPbIqV92AaeXatLxBI9gBaebbnrfifHhDYfgasaacH8akY=wiFfYdH8Gipec8Eeeu0xXdbba9frFj0=OqFfea0dXdd9vqai=hGuQ8kuc9pgc9s8qqaq=dirpe0xb9q8qiLsFr0=vr0=vr0dc8meaabaqaciaacaGaaeqabaqabeGadaaakeaacqWG5bqEdaWgaaWcbaacciGae83Xdm2aaSbaaWqaaiabiodaZiabcYcaSiab=T7aSnaaBaaabaGaemOyaigabeaaaeqaaaWcbeaakiabgsMiJkabigdaXaaa@37E9@	Line sweep at *b*_*t *_= 9
yχ2,λb+yχ3,λb≤1 MathType@MTEF@5@5@+=feaafiart1ev1aaatCvAUfKttLearuWrP9MDH5MBPbIqV92AaeXatLxBI9gBaebbnrfifHhDYfgasaacH8akY=wiFfYdH8Gipec8Eeeu0xXdbba9frFj0=OqFfea0dXdd9vqai=hGuQ8kuc9pgc9s8qqaq=dirpe0xb9q8qiLsFr0=vr0=vr0dc8meaabaqaciaacaGaaeqabaqabeGadaaakeaacqWG5bqEdaWgaaWcbaacciGae83Xdm2aaSbaaWqaaiabikdaYiabcYcaSiab=T7aSnaaBaaabaGaemOyaigabeaaaeqaaaWcbeaakiabgUcaRiabdMha5naaBaaaleaacqWFhpWydaWgaaadbaGaeG4mamJaeiilaWIae83UdW2aaSbaaeaacqWGIbGyaeqaaaqabaaaleqaaOGaeyizImQaeGymaedaaa@4155@	Line sweep at *b*_*t *_= 12
yχ2,λb≤1 MathType@MTEF@5@5@+=feaafiart1ev1aaatCvAUfKttLearuWrP9MDH5MBPbIqV92AaeXatLxBI9gBaebbnrfifHhDYfgasaacH8akY=wiFfYdH8Gipec8Eeeu0xXdbba9frFj0=OqFfea0dXdd9vqai=hGuQ8kuc9pgc9s8qqaq=dirpe0xb9q8qiLsFr0=vr0=vr0dc8meaabaqaciaacaGaaeqabaqabeGadaaakeaacqWG5bqEdaWgaaWcbaacciGae83Xdm2aaSbaaWqaaiabikdaYiabcYcaSiab=T7aSnaaBaaabaGaemOyaigabeaaaeqaaaWcbeaakiabgsMiJkabigdaXaaa@37E7@	Line sweep at *b*_*t *_= 13
yχ5,λb≤1 MathType@MTEF@5@5@+=feaafiart1ev1aaatCvAUfKttLearuWrP9MDH5MBPbIqV92AaeXatLxBI9gBaebbnrfifHhDYfgasaacH8akY=wiFfYdH8Gipec8Eeeu0xXdbba9frFj0=OqFfea0dXdd9vqai=hGuQ8kuc9pgc9s8qqaq=dirpe0xb9q8qiLsFr0=vr0=vr0dc8meaabaqaciaacaGaaeqabaqabeGadaaakeaacqWG5bqEdaWgaaWcbaacciGae83Xdm2aaSbaaWqaaiabiwda1iabcYcaSiab=T7aSnaaBaaabaGaemOyaigabeaaaeqaaaWcbeaakiabgsMiJkabigdaXaaa@37ED@	Line sweep at *b*_*t *_= 14
Consistency inequalities:	**(4,5)**
yχ1,λa−xχ1≥0 MathType@MTEF@5@5@+=feaafiart1ev1aaatCvAUfKttLearuWrP9MDH5MBPbIqV92AaeXatLxBI9gBaebbnrfifHhDYfgasaacH8akY=wiFfYdH8Gipec8Eeeu0xXdbba9frFj0=OqFfea0dXdd9vqai=hGuQ8kuc9pgc9s8qqaq=dirpe0xb9q8qiLsFr0=vr0=vr0dc8meaabaqaciaacaGaaeqabaqabeGadaaakeaacqWG5bqEdaWgaaWcbaacciGae83Xdm2aaSbaaWqaaiabigdaXiabcYcaSiab=T7aSnaaBaaabaGaemyyaegabeaaaeqaaaWcbeaakiabgkHiTiabdIha4naaBaaaleaacqWFhpWydaWgaaadbaGaeGymaedabeaaaSqabaGccqGHLjYScqaIWaamaaa@3D68@	yχ1,λb−xχ1≥0 MathType@MTEF@5@5@+=feaafiart1ev1aaatCvAUfKttLearuWrP9MDH5MBPbIqV92AaeXatLxBI9gBaebbnrfifHhDYfgasaacH8akY=wiFfYdH8Gipec8Eeeu0xXdbba9frFj0=OqFfea0dXdd9vqai=hGuQ8kuc9pgc9s8qqaq=dirpe0xb9q8qiLsFr0=vr0=vr0dc8meaabaqaciaacaGaaeqabaqabeGadaaakeaacqWG5bqEdaWgaaWcbaacciGae83Xdm2aaSbaaWqaaiabigdaXiabcYcaSiab=T7aSnaaBaaabaGaemOyaigabeaaaeqaaaWcbeaakiabgkHiTiabdIha4naaBaaaleaacqWFhpWydaWgaaadbaGaeGymaedabeaaaSqabaGccqGHLjYScqaIWaamaaa@3D6A@
yχ2,λa−xχ2≥0 MathType@MTEF@5@5@+=feaafiart1ev1aaatCvAUfKttLearuWrP9MDH5MBPbIqV92AaeXatLxBI9gBaebbnrfifHhDYfgasaacH8akY=wiFfYdH8Gipec8Eeeu0xXdbba9frFj0=OqFfea0dXdd9vqai=hGuQ8kuc9pgc9s8qqaq=dirpe0xb9q8qiLsFr0=vr0=vr0dc8meaabaqaciaacaGaaeqabaqabeGadaaakeaacqWG5bqEdaWgaaWcbaacciGae83Xdm2aaSbaaWqaaiabikdaYiabcYcaSiab=T7aSnaaBaaabaGaemyyaegabeaaaeqaaaWcbeaakiabgkHiTiabdIha4naaBaaaleaacqWFhpWydaWgaaadbaGaeGOmaidabeaaaSqabaGccqGHLjYScqaIWaamaaa@3D6C@	yχ2,λb−xχ2≥0 MathType@MTEF@5@5@+=feaafiart1ev1aaatCvAUfKttLearuWrP9MDH5MBPbIqV92AaeXatLxBI9gBaebbnrfifHhDYfgasaacH8akY=wiFfYdH8Gipec8Eeeu0xXdbba9frFj0=OqFfea0dXdd9vqai=hGuQ8kuc9pgc9s8qqaq=dirpe0xb9q8qiLsFr0=vr0=vr0dc8meaabaqaciaacaGaaeqabaqabeGadaaakeaacqWG5bqEdaWgaaWcbaacciGae83Xdm2aaSbaaWqaaiabikdaYiabcYcaSiab=T7aSnaaBaaabaGaemOyaigabeaaaeqaaaWcbeaakiabgkHiTiabdIha4naaBaaaleaacqWFhpWydaWgaaadbaGaeGOmaidabeaaaSqabaGccqGHLjYScqaIWaamaaa@3D6E@
yχ3,λa−xχ3≥0 MathType@MTEF@5@5@+=feaafiart1ev1aaatCvAUfKttLearuWrP9MDH5MBPbIqV92AaeXatLxBI9gBaebbnrfifHhDYfgasaacH8akY=wiFfYdH8Gipec8Eeeu0xXdbba9frFj0=OqFfea0dXdd9vqai=hGuQ8kuc9pgc9s8qqaq=dirpe0xb9q8qiLsFr0=vr0=vr0dc8meaabaqaciaacaGaaeqabaqabeGadaaakeaacqWG5bqEdaWgaaWcbaacciGae83Xdm2aaSbaaWqaaiabiodaZiabcYcaSiab=T7aSnaaBaaabaGaemyyaegabeaaaeqaaaWcbeaakiabgkHiTiabdIha4naaBaaaleaacqWFhpWydaWgaaadbaGaeG4mamdabeaaaSqabaGccqGHLjYScqaIWaamaaa@3D70@	yχ3,λb−xχ3≥0 MathType@MTEF@5@5@+=feaafiart1ev1aaatCvAUfKttLearuWrP9MDH5MBPbIqV92AaeXatLxBI9gBaebbnrfifHhDYfgasaacH8akY=wiFfYdH8Gipec8Eeeu0xXdbba9frFj0=OqFfea0dXdd9vqai=hGuQ8kuc9pgc9s8qqaq=dirpe0xb9q8qiLsFr0=vr0=vr0dc8meaabaqaciaacaGaaeqabaqabeGadaaakeaacqWG5bqEdaWgaaWcbaacciGae83Xdm2aaSbaaWqaaiabiodaZiabcYcaSiab=T7aSnaaBaaabaGaemOyaigabeaaaeqaaaWcbeaakiabgkHiTiabdIha4naaBaaaleaacqWFhpWydaWgaaadbaGaeG4mamdabeaaaSqabaGccqGHLjYScqaIWaamaaa@3D72@
yχ4,λa−xχ4≥0 MathType@MTEF@5@5@+=feaafiart1ev1aaatCvAUfKttLearuWrP9MDH5MBPbIqV92AaeXatLxBI9gBaebbnrfifHhDYfgasaacH8akY=wiFfYdH8Gipec8Eeeu0xXdbba9frFj0=OqFfea0dXdd9vqai=hGuQ8kuc9pgc9s8qqaq=dirpe0xb9q8qiLsFr0=vr0=vr0dc8meaabaqaciaacaGaaeqabaqabeGadaaakeaacqWG5bqEdaWgaaWcbaacciGae83Xdm2aaSbaaWqaaiabisda0iabcYcaSiab=T7aSnaaBaaabaGaemyyaegabeaaaeqaaaWcbeaakiabgkHiTiabdIha4naaBaaaleaacqWFhpWydaWgaaadbaGaeGinaqdabeaaaSqabaGccqGHLjYScqaIWaamaaa@3D74@	yχ4,λb−xχ4≥0 MathType@MTEF@5@5@+=feaafiart1ev1aaatCvAUfKttLearuWrP9MDH5MBPbIqV92AaeXatLxBI9gBaebbnrfifHhDYfgasaacH8akY=wiFfYdH8Gipec8Eeeu0xXdbba9frFj0=OqFfea0dXdd9vqai=hGuQ8kuc9pgc9s8qqaq=dirpe0xb9q8qiLsFr0=vr0=vr0dc8meaabaqaciaacaGaaeqabaqabeGadaaakeaacqWG5bqEdaWgaaWcbaacciGae83Xdm2aaSbaaWqaaiabisda0iabcYcaSiab=T7aSnaaBaaabaGaemOyaigabeaaaeqaaaWcbeaakiabgkHiTiabdIha4naaBaaaleaacqWFhpWydaWgaaadbaGaeGinaqdabeaaaSqabaGccqGHLjYScqaIWaamaaa@3D76@
yχ5,λa−xχ5≥0 MathType@MTEF@5@5@+=feaafiart1ev1aaatCvAUfKttLearuWrP9MDH5MBPbIqV92AaeXatLxBI9gBaebbnrfifHhDYfgasaacH8akY=wiFfYdH8Gipec8Eeeu0xXdbba9frFj0=OqFfea0dXdd9vqai=hGuQ8kuc9pgc9s8qqaq=dirpe0xb9q8qiLsFr0=vr0=vr0dc8meaabaqaciaacaGaaeqabaqabeGadaaakeaacqWG5bqEdaWgaaWcbaacciGae83Xdm2aaSbaaWqaaiabiwda1iabcYcaSiab=T7aSnaaBaaabaGaemyyaegabeaaaeqaaaWcbeaakiabgkHiTiabdIha4naaBaaaleaacqWFhpWydaWgaaadbaGaeGynaudabeaaaSqabaGccqGHLjYScqaIWaamaaa@3D78@	yχ5,λb−xχ5≥0 MathType@MTEF@5@5@+=feaafiart1ev1aaatCvAUfKttLearuWrP9MDH5MBPbIqV92AaeXatLxBI9gBaebbnrfifHhDYfgasaacH8akY=wiFfYdH8Gipec8Eeeu0xXdbba9frFj0=OqFfea0dXdd9vqai=hGuQ8kuc9pgc9s8qqaq=dirpe0xb9q8qiLsFr0=vr0=vr0dc8meaabaqaciaacaGaaeqabaqabeGadaaakeaacqWG5bqEdaWgaaWcbaacciGae83Xdm2aaSbaaWqaaiabiwda1iabcYcaSiab=T7aSnaaBaaabaGaemOyaigabeaaaeqaaaWcbeaakiabgkHiTiabdIha4naaBaaaleaacqWFhpWydaWgaaadbaGaeGynaudabeaaaSqabaGccqGHLjYScqaIWaamaaa@3D7A@

The constraints of the conflict graph for the set of fragments in Figure 6c.

#### Basic Definitions and Notations

The following definitions/notations are used uniformly throughout the paper unless otherwise stated:

• Protein structures are denoted by *S*_*a*_, *S*_*b*_,....

• A substructure λi,ka
 MathType@MTEF@5@5@+=feaafiart1ev1aaatCvAUfKttLearuWrP9MDH5MBPbIqV92AaeXatLxBI9gBaebbnrfifHhDYfgasaacH8akY=wiFfYdH8Gipec8Eeeu0xXdbba9frFj0=OqFfea0dXdd9vqai=hGuQ8kuc9pgc9s8qqaq=dirpe0xb9q8qiLsFr0=vr0=vr0dc8meaabaqaciaacaGaaeqabaqabeGadaaakeaaiiGacqWF7oaBdaqhaaWcbaGaemyAaKMaeiilaWIaem4AaSgabaGaemyyaegaaaaa@3379@ of a protein structure *S*_*a *_is a continuous fragment λi,ka
 MathType@MTEF@5@5@+=feaafiart1ev1aaatCvAUfKttLearuWrP9MDH5MBPbIqV92AaeXatLxBI9gBaebbnrfifHhDYfgasaacH8akY=wiFfYdH8Gipec8Eeeu0xXdbba9frFj0=OqFfea0dXdd9vqai=hGuQ8kuc9pgc9s8qqaq=dirpe0xb9q8qiLsFr0=vr0=vr0dc8meaabaqaciaacaGaaeqabaqabeGadaaakeaaiiGacqWF7oaBdaqhaaWcbaGaemyAaKMaeiilaWIaem4AaSgabaGaemyyaegaaaaa@3379@, where *i *is the residue index of the beginning of the substructure and *k *is the length (number of residues) of the substructure. We will denote such a substructure simply by *λ*^*a *^if *i *and *k *are clear from the context or irrelevant.

• A residue *a*_*t *_∈ *S*_*a *_is a *part *of a substructure λi,ka
 MathType@MTEF@5@5@+=feaafiart1ev1aaatCvAUfKttLearuWrP9MDH5MBPbIqV92AaeXatLxBI9gBaebbnrfifHhDYfgasaacH8akY=wiFfYdH8Gipec8Eeeu0xXdbba9frFj0=OqFfea0dXdd9vqai=hGuQ8kuc9pgc9s8qqaq=dirpe0xb9q8qiLsFr0=vr0=vr0dc8meaabaqaciaacaGaaeqabaqabeGadaaakeaaiiGacqWF7oaBdaqhaaWcbaGaemyAaKMaeiilaWIaem4AaSgabaGaemyyaegaaaaa@3379@ if *i *≤ *t *≤ *i *+ *k *- 1.

• Λ_*a *_is the set of all continuous substructures or fragments of protein structure *S*_*a *_that is under consideration in our algorithm.

• *χ*_*i, j, k *_(or simply *χ *when the other parameters are understood from the context) denotes an ordered pair (λi,ka,λj,kb
 MathType@MTEF@5@5@+=feaafiart1ev1aaatCvAUfKttLearuWrP9MDH5MBPbIqV92AaeXatLxBI9gBaebbnrfifHhDYfgasaacH8akY=wiFfYdH8Gipec8Eeeu0xXdbba9frFj0=OqFfea0dXdd9vqai=hGuQ8kuc9pgc9s8qqaq=dirpe0xb9q8qiLsFr0=vr0=vr0dc8meaabaqaciaacaGaaeqabaqabeGadaaakeaaiiGacqWF7oaBdaqhaaWcbaGaemyAaKMaeiilaWIaem4AaSgabaGaemyyaegaaOGaeiilaWIae83UdW2aa0baaSqaaiabdQgaQjabcYcaSiabdUgaRbqaaiabdkgaIbaaaaa@3B28@) of equal length substructures of two protein structures *S*_*a *_and *S*_*b*_.

• Two ordered pairs of substructures (λi,ka,λj,kb
 MathType@MTEF@5@5@+=feaafiart1ev1aaatCvAUfKttLearuWrP9MDH5MBPbIqV92AaeXatLxBI9gBaebbnrfifHhDYfgasaacH8akY=wiFfYdH8Gipec8Eeeu0xXdbba9frFj0=OqFfea0dXdd9vqai=hGuQ8kuc9pgc9s8qqaq=dirpe0xb9q8qiLsFr0=vr0=vr0dc8meaabaqaciaacaGaaeqabaqabeGadaaakeaaiiGacqWF7oaBdaqhaaWcbaGaemyAaKMaeiilaWIaem4AaSgabaGaemyyaegaaOGaeiilaWIae83UdW2aa0baaSqaaiabdQgaQjabcYcaSiabdUgaRbqaaiabdkgaIbaaaaa@3B28@) and (λi′,k′a,λj′,k′b
 MathType@MTEF@5@5@+=feaafiart1ev1aaatCvAUfKttLearuWrP9MDH5MBPbIqV92AaeXatLxBI9gBaebbnrfifHhDYfgasaacH8akY=wiFfYdH8Gipec8Eeeu0xXdbba9frFj0=OqFfea0dXdd9vqai=hGuQ8kuc9pgc9s8qqaq=dirpe0xb9q8qiLsFr0=vr0=vr0dc8meaabaqaciaacaGaaeqabaqabeGadaaakeaaiiGacqWF7oaBdaqhaaWcbaGafmyAaKMbauaacqGGSaalcuWGRbWAgaqbaaqaaiabdggaHbaakiabcYcaSiab=T7aSnaaDaaaleaacuWGQbGAgaqbaiabcYcaSiqbdUgaRzaafaaabaGaemOyaigaaaaa@3B58@) are called *inconsistent *if and only if at least one of the pairs of substructures {λi,ka,λi′,k′a
 MathType@MTEF@5@5@+=feaafiart1ev1aaatCvAUfKttLearuWrP9MDH5MBPbIqV92AaeXatLxBI9gBaebbnrfifHhDYfgasaacH8akY=wiFfYdH8Gipec8Eeeu0xXdbba9frFj0=OqFfea0dXdd9vqai=hGuQ8kuc9pgc9s8qqaq=dirpe0xb9q8qiLsFr0=vr0=vr0dc8meaabaqaciaacaGaaeqabaqabeGadaaakeaaiiGacqWF7oaBdaqhaaWcbaGaemyAaKMaeiilaWIaem4AaSgabaGaemyyaegaaOGaeiilaWIae83UdW2aa0baaSqaaiqbdMgaPzaafaGaeiilaWIafm4AaSMbauaaaeaacqWGHbqyaaaaaa@3B3C@} and {λj,ka,λj′,k′a
 MathType@MTEF@5@5@+=feaafiart1ev1aaatCvAUfKttLearuWrP9MDH5MBPbIqV92AaeXatLxBI9gBaebbnrfifHhDYfgasaacH8akY=wiFfYdH8Gipec8Eeeu0xXdbba9frFj0=OqFfea0dXdd9vqai=hGuQ8kuc9pgc9s8qqaq=dirpe0xb9q8qiLsFr0=vr0=vr0dc8meaabaqaciaacaGaaeqabaqabeGadaaakeaaiiGacqWF7oaBdaqhaaWcbaGaemOAaOMaeiilaWIaem4AaSgabaGaemyyaegaaOGaeiilaWIae83UdW2aa0baaSqaaiqbdQgaQzaafaGaeiilaWIafm4AaSMbauaaaeaacqWGHbqyaaaaaa@3B40@} are not disjoint.

We are now ready to formalize our substructure similarity identification problem as below:

**Problem name: **Basic Substructure Similarity Identification (BSSI_Λ, *σ*_).

**Instance: **a set Λ = {*χ*_*i, j, k*_|*i, j, k *∈ ℕ} ⊂ Λ_*a *_× Λ_*b *_of ordered pairs of equal length substructures of *S*_*a *_and *S*_*b *_and a similarity function *σ *: Λ ↦ ℝ^+ ^mapping each pair of substructures to a positive similarity value.

**Valid Solutions: **a set of substructure pairs {χi1,j1,k1,χi2,j2,k2,...χit,jt,kt
 MathType@MTEF@5@5@+=feaafiart1ev1aaatCvAUfKttLearuWrP9MDH5MBPbIqV92AaeXatLxBI9gBaebbnrfifHhDYfgasaacH8akY=wiFfYdH8Gipec8Eeeu0xXdbba9frFj0=OqFfea0dXdd9vqai=hGuQ8kuc9pgc9s8qqaq=dirpe0xb9q8qiLsFr0=vr0=vr0dc8meaabaqaciaacaGaaeqabaqabeGadaaakeaaiiGacqWFhpWydaWgaaWcbaGaemyAaK2aaSbaaWqaaiabigdaXaqabaWccqGGSaalcqWGQbGAdaWgaaadbaGaeGymaedabeaaliabcYcaSiabdUgaRnaaBaaameaacqaIXaqmaeqaaaWcbeaakiabcYcaSiab=D8aJnaaBaaaleaacqWGPbqAdaWgaaadbaGaeGOmaidabeaaliabcYcaSiabdQgaQnaaBaaameaacqaIYaGmaeqaaSGaeiilaWIaem4AaS2aaSbaaWqaaiabikdaYaqabaaaleqaaOGaeiilaWIaeiOla4IaeiOla4IaeiOla4Iae83Xdm2aaSbaaSqaaiabdMgaPnaaBaaameaacqWG0baDaeqaaSGaeiilaWIaemOAaO2aaSbaaWqaaiabdsha0bqabaWccqGGSaalcqWGRbWAdaWgaaadbaGaemiDaqhabeaaaSqabaaaaa@5448@} that are mutually consistent.

**Objective: ***maximize *the total similarity of the selection ∑ℓ=1tσ(χiℓ,jℓ,kℓ)
 MathType@MTEF@5@5@+=feaafiart1ev1aaatCvAUfKttLearuWrP9MDH5MBPbIqV92AaeXatLxBI9gBaebbnrfifHhDYfgasaacH8akY=wiFfYdH8Gipec8Eeeu0xXdbba9frFj0=OqFfea0dXdd9vqai=hGuQ8kuc9pgc9s8qqaq=dirpe0xb9q8qiLsFr0=vr0=vr0dc8meaabaqaciaacaGaaeqabaqabeGadaaakeaadaaeWaqaaGGaciab=n8aZjabcIcaOiab=D8aJnaaBaaaleaacqWGPbqAdaWgaaadbaGaeS4eHWgabeaaliabcYcaSiabdQgaQnaaBaaameaacqWItecBaeqaaSGaeiilaWIaem4AaS2aaSbaaWqaaiabloriSbqabaaaleqaaOGaeiykaKcaleaacqWItecBcqGH9aqpcqaIXaqmaeaacqWG0baDa0GaeyyeIuoaaaa@42BC@.

#### An Algorithm Based on the Local-Ratio Approach

The *BSSI*_Λ, *σ *_problem is a special case of the well-known maximum weight independent set problem in graph theory [33]. In fact, *BSSI*_Λ, *σ *_itself is MAX-SNP-hard (i. e., there is a constant 0 <*ε *< 1 such that no polynomial-time algorithm can return a solution with a value of the objective function that is within 1 - *ε *times the optimum [34] unless P = NP) even when all the substructures are restricted to have lengths at most 2 [32, Theorem 2.1]. Our approach is to adopt the approximation algorithm for scheduling split-interval graphs [32] which itself is based on a fractional version of the local-ratio approach. For ease in description of our algorithm, we introduce the following definitions.

**Definition 1 ***For any subset*, Δ ⊆ Λ *the conflict graph G*_Δ _= (*V*_Δ_, *E*_Δ_) *is the graph in which V*_Δ _= {*χ*|*χ *∈ Δ} *and E*_Δ _= {{*χ*, *χ'*}|*χ*, *χ'*, ∈ Δ *and the pair *{*χ*, *χ*'} *is not consistent*}

**Definition 2 ***The closed neighborhood Nbr*_Δ _[*χ*] *of a vertex χ of G*_Δ _*is *{*χ*' | {*χ*, *χ*' } ∈ *E*_Δ_} ∪ {*χ*}.

For an instance of *BSSI*_Λ,*σ *_with Δ ⊆ Λ we introduce three types of indicator variables as follows. For every *χ *= (λ_*a*_, λ_*b*_) ∈ Δ, we introduce three indicator variables *x*_*χ*_, yχλa
 MathType@MTEF@5@5@+=feaafiart1ev1aaatCvAUfKttLearuWrP9MDH5MBPbIqV92AaeXatLxBI9gBaebbnrfifHhDYfgasaacH8akY=wiFfYdH8Gipec8Eeeu0xXdbba9frFj0=OqFfea0dXdd9vqai=hGuQ8kuc9pgc9s8qqaq=dirpe0xb9q8qiLsFr0=vr0=vr0dc8meaabaqaciaacaGaaeqabaqabeGadaaakeaacqWG5bqEdaWgaaWcbaacciGae83Xdm2aaSbaaWqaaiab=T7aSnaaBaaabaGaemyyaegabeaaaeqaaaWcbeaaaaa@3364@ and yχλb
 MathType@MTEF@5@5@+=feaafiart1ev1aaatCvAUfKttLearuWrP9MDH5MBPbIqV92AaeXatLxBI9gBaebbnrfifHhDYfgasaacH8akY=wiFfYdH8Gipec8Eeeu0xXdbba9frFj0=OqFfea0dXdd9vqai=hGuQ8kuc9pgc9s8qqaq=dirpe0xb9q8qiLsFr0=vr0=vr0dc8meaabaqaciaacaGaaeqabaqabeGadaaakeaacqWG5bqEdaWgaaWcbaacciGae83Xdm2aaSbaaWqaaiab=T7aSnaaBaaabaGaemOyaigabeaaaeqaaaWcbeaaaaa@3366@ ∈ {0,1}. *x*_*χ *_indicates whether the substructure pair should be used (*x*_*χ *_= 1) or not (*x*_*χ *_= 0) in the final alignment. yχλa
 MathType@MTEF@5@5@+=feaafiart1ev1aaatCvAUfKttLearuWrP9MDH5MBPbIqV92AaeXatLxBI9gBaebbnrfifHhDYfgasaacH8akY=wiFfYdH8Gipec8Eeeu0xXdbba9frFj0=OqFfea0dXdd9vqai=hGuQ8kuc9pgc9s8qqaq=dirpe0xb9q8qiLsFr0=vr0=vr0dc8meaabaqaciaacaGaaeqabaqabeGadaaakeaacqWG5bqEdaWgaaWcbaacciGae83Xdm2aaSbaaWqaaiab=T7aSnaaBaaabaGaemyyaegabeaaaeqaaaWcbeaaaaa@3364@ and yχλb
 MathType@MTEF@5@5@+=feaafiart1ev1aaatCvAUfKttLearuWrP9MDH5MBPbIqV92AaeXatLxBI9gBaebbnrfifHhDYfgasaacH8akY=wiFfYdH8Gipec8Eeeu0xXdbba9frFj0=OqFfea0dXdd9vqai=hGuQ8kuc9pgc9s8qqaq=dirpe0xb9q8qiLsFr0=vr0=vr0dc8meaabaqaciaacaGaaeqabaqabeGadaaakeaacqWG5bqEdaWgaaWcbaacciGae83Xdm2aaSbaaWqaaiab=T7aSnaaBaaabaGaemOyaigabeaaaeqaaaWcbeaaaaa@3366@ are artificial selection variables for *λ*_*a *_and *λ*_*b *_that allows us to encode consistency in the selected substructures in a way that guarantees good approximation bounds. Our algorithm for solving an instance of *BSSI*_Λ,*σ *_can now be described as follows. We initialize Δ = Λ. Then, the following algorithm is executed:

1. Solve a linear programming (LP) formulation of *BSSI*_Λ,*σ *_by relaxing a corresponding integer programming version of the *BSSI*_Λ,*σ *_problem.

maximize

∑χ∈Δσ(χ)⋅xχ     (1)
 MathType@MTEF@5@5@+=feaafiart1ev1aaatCvAUfKttLearuWrP9MDH5MBPbIqV92AaeXatLxBI9gBaebbnrfifHhDYfgasaacH8akY=wiFfYdH8Gipec8Eeeu0xXdbba9frFj0=OqFfea0dXdd9vqai=hGuQ8kuc9pgc9s8qqaq=dirpe0xb9q8qiLsFr0=vr0=vr0dc8meaabaqaciaacaGaaeqabaqabeGadaaakeaadaaeqbqaaGGaciab=n8aZjabcIcaOiab=D8aJjabcMcaPiabgwSixlabdIha4naaBaaaleaacqWFhpWyaeqaaaqaaiab=D8aJjabgIGiolabfs5aebqab0GaeyyeIuoaaaa@3E2F@

Subject to

∑at∈λa∈Λayχλa≤1∀at∈Sa     (2)
 MathType@MTEF@5@5@+=feaafiart1ev1aaatCvAUfKttLearuWrP9MDH5MBPbIqV92AaeXatLxBI9gBaebbnrfifHhDYfgasaacH8akY=wiFfYdH8Gipec8Eeeu0xXdbba9frFj0=OqFfea0dXdd9vqai=hGuQ8kuc9pgc9s8qqaq=dirpe0xb9q8qiLsFr0=vr0=vr0dc8meaabaqaciaacaGaaeqabaqabeGadaaakeaafaqabeqadaaabaWaaabuaeaacqWG5bqEdaWgaaWcbaacciGae83Xdm2aaSbaaWqaaiab=T7aSnaaBaaabaGaemyyaegabeaaaeqaaaWcbeaaaeaacqWGHbqydaWgaaadbaGaemiDaqhabeaaliabgIGiolab=T7aSnaaCaaameqabaGaemyyaegaaSGaeyicI4Saeu4MdW0aaSbaaWqaaiabdggaHbqabaaaleqaniabggHiLdaakeaacqGHKjYOcqaIXaqmaeaacqGHaiIicqWGHbqydaWgaaWcbaGaemiDaqhabeaakiabgIGiolabdofatnaaBaaaleaacqWGHbqyaeqaaaaaaaa@4C4D@

∑at∈λb∈Λbyχλb≤1∀at∈Sb     (3)
 MathType@MTEF@5@5@+=feaafiart1ev1aaatCvAUfKttLearuWrP9MDH5MBPbIqV92AaeXatLxBI9gBaebbnrfifHhDYfgasaacH8akY=wiFfYdH8Gipec8Eeeu0xXdbba9frFj0=OqFfea0dXdd9vqai=hGuQ8kuc9pgc9s8qqaq=dirpe0xb9q8qiLsFr0=vr0=vr0dc8meaabaqaciaacaGaaeqabaqabeGadaaakeaafaqabeqadaaabaWaaabuaeaacqWG5bqEdaWgaaWcbaacciGae83Xdm2aaSbaaWqaaiabeU7aSnaaBaaabaGaemOyaigabeaaaeqaaaWcbeaaaeaacqWGHbqydaWgaaadbaGaemiDaqhabeaaliabgIGiolab=T7aSnaaCaaameqabaGaemOyaigaaSGaeyicI4Saeu4MdW0aaSbaaWqaaiabdkgaIbqabaaaleqaniabggHiLdaakeaacqGHKjYOcqaIXaqmaeaacqGHaiIicqWGHbqydaWgaaWcbaGaemiDaqhabeaakiabgIGiolabdofatnaaBaaaleaacqWGIbGyaeqaaaaaaaa@4C5A@

yχλa−xχ≥0∀χ∈Δ     (4)
 MathType@MTEF@5@5@+=feaafiart1ev1aaatCvAUfKttLearuWrP9MDH5MBPbIqV92AaeXatLxBI9gBaebbnrfifHhDYfgasaacH8akY=wiFfYdH8Gipec8Eeeu0xXdbba9frFj0=OqFfea0dXdd9vqai=hGuQ8kuc9pgc9s8qqaq=dirpe0xb9q8qiLsFr0=vr0=vr0dc8meaabaqaciaacaGaaeqabaqabeGadaaakeaafaqabeqadaaabaGaemyEaK3aaSbaaSqaaGGaciab=D8aJnaaBaaameaacqWF7oaBdaWgaaqaaiabdggaHbqabaaabeaaaSqabaGccqGHsislcqWG4baEdaWgaaWcbaGae83XdmgabeaaaOqaaiabgwMiZkabicdaWaqaaiabgcGiIiab=D8aJjabgIGiolabfs5aebaaaaa@3FEB@

yχλb−xχ≥0∀χ∈Δ     (5)
 MathType@MTEF@5@5@+=feaafiart1ev1aaatCvAUfKttLearuWrP9MDH5MBPbIqV92AaeXatLxBI9gBaebbnrfifHhDYfgasaacH8akY=wiFfYdH8Gipec8Eeeu0xXdbba9frFj0=OqFfea0dXdd9vqai=hGuQ8kuc9pgc9s8qqaq=dirpe0xb9q8qiLsFr0=vr0=vr0dc8meaabaqaciaacaGaaeqabaqabeGadaaakeaafaqabeqadaaabaGaemyEaK3aaSbaaSqaaGGaciab=D8aJnaaBaaameaacqWF7oaBdaWgaaqaaiabdkgaIbqabaaabeaaaSqabaGccqGHsislcqWG4baEdaWgaaWcbaGae83XdmgabeaaaOqaaiabgwMiZkabicdaWaqaaiabgcGiIiab=D8aJjabgIGiolabfs5aebaaaaa@3FED@

xχ,yχλa,yχλb≥0∀χ∈Δ     (6)
 MathType@MTEF@5@5@+=feaafiart1ev1aaatCvAUfKttLearuWrP9MDH5MBPbIqV92AaeXatLxBI9gBaebbnrfifHhDYfgasaacH8akY=wiFfYdH8Gipec8Eeeu0xXdbba9frFj0=OqFfea0dXdd9vqai=hGuQ8kuc9pgc9s8qqaq=dirpe0xb9q8qiLsFr0=vr0=vr0dc8meaabaqaciaacaGaaeqabaqabeGadaaakeaafaqabeqadaaabaGaemiEaG3aaSbaaSqaaGGaciab=D8aJbqabaGccqGGSaalcqWG5bqEdaWgaaWcbaGae83Xdm2aaSbaaWqaaiab=T7aSnaaBaaabaGaemyyaegabeaaaeqaaaWcbeaakiabcYcaSiabdMha5naaBaaaleaacqWFhpWydaWgaaadbaGae83UdW2aaSbaaeaacqWGIbGyaeqaaaqabaaaleqaaaGcbaGaeyyzImRaeGimaadabaGaeyiaIiIae83XdmMaeyicI4SaeuiLdqeaaaaa@4776@

2. For every vertex *χ *∈ *V*_Δ _of *G*_Δ_, compute its *local conflict number *αχ=∑χ′∈NbrΔ[χ]xχ′
 MathType@MTEF@5@5@+=feaafiart1ev1aaatCvAUfKttLearuWrP9MDH5MBPbIqV92AaeXatLxBI9gBaebbnrfifHhDYfgasaacH8akY=wiFfYdH8Gipec8Eeeu0xXdbba9frFj0=OqFfea0dXdd9vqai=hGuQ8kuc9pgc9s8qqaq=dirpe0xb9q8qiLsFr0=vr0=vr0dc8meaabaqaciaacaGaaeqabaqabeGadaaakeaaiiGacqWFXoqydaWgaaWcbaGae83Xdmgabeaakiabg2da9maaqababaGaemiEaG3aaSbaaSqaaiqb=D8aJzaafaaabeaaaeaacuWFhpWygaqbaiabgIGiolabb6eaojabbkgaIjabbkhaYnaaBaaameaacqqHuoaraeqaaSGaei4waSLae83XdmMaeiyxa0fabeqdcqGHris5aaaa@4366@. Let *χ*_*min *_be the vertex with the *minimum *local conflict number. Define a new similarity function *σ*_*new *_from *σ *as follows:

σnew(χ)={σ(χ)if χ∉NbrΔ[χmin]σ(χ)−σ(χmin)otherwise
 MathType@MTEF@5@5@+=feaafiart1ev1aaatCvAUfKttLearuWrP9MDH5MBPbIqV92AaeXatLxBI9gBaebbnrfifHhDYfgasaacH8akY=wiFfYdH8Gipec8Eeeu0xXdbba9frFj0=OqFfea0dXdd9vqai=hGuQ8kuc9pgc9s8qqaq=dirpe0xb9q8qiLsFr0=vr0=vr0dc8meaabaqaciaacaGaaeqabaqabeGadaaakeaaiiGacqWFdpWCdaWgaaWcbaGaemOBa4MaemyzauMaem4DaChabeaakiabcIcaOiab=D8aJjabcMcaPiabg2da9maaceqabaqbaeaabiGaaaqaaiab=n8aZjabcIcaOiab=D8aJjabcMcaPaqaaiabbMgaPjabbAgaMjabbccaGiab=D8aJjabgMGiplabb6eaojabbkgaIjabbkhaYnaaBaaaleaacqqHuoaraeqaaOGaei4waSLae83Xdm2aaSbaaSqaaiabd2gaTjabdMgaPjabd6gaUbqabaGccqGGDbqxaeaacqWFdpWCcqGGOaakcqWFhpWycqGGPaqkcqGHsislcqWFdpWCcqGGOaakcqWFhpWydaWgaaWcbaGaemyBa0MaemyAaKMaemOBa4gabeaakiabcMcaPaqaaiabb+gaVjabbsha0jabbIgaOjabbwgaLjabbkhaYjabbEha3jabbMgaPjabbohaZjabbwgaLbaaaiaawUhaaaaa@6E39@

3. Create Δ_*new *_⊆ Δ by removing from Δ every substructure pair *χ *such that *σ*_*new*_(*χ*) ≤ 0. Push each removed substructure on to a stack in arbitrary order.

4. If Δ_*new *_≠ ∅ then repeat from step 1 setting Δ = Δ_*new *_and *σ *= *σ*_*new*_. Otherwise, continue to step 5.

5. Repeatedly pop the stack, adding the substructure pair to the alignment as long as the following conditions are met:

(a) The substructure pair is consistent with all other substructure pairs that already exist in the selection.

(b) The *cRMSD *of the alignment does not change by a threshold. This condition bridges the gap between optimizing a local similarity between substructures and optimizing the tertiary similarity of the alignment by guaranteeing that each substructure from a substructure pair is in the same spatial arrangement in the global alignment.

A brief intuitive explanation of the various inequalities in the LP formulation as described above in terms of their original integer programming formulation is as follows:

• The "interval clique" inequalities in Equation (2) (resp. Equation (3)) ensure that the various substructures of *S*_*a *_(resp. *S*_*b*_) in the selected substructure pairs from Δ are mutually disjoint.

• Inequalities in Equation 4 and Equation 5 ensure consistencies between the indicator variable for each substructure pair *χ *and its two substructures λ_*a *_and λ_*b*_.

• Inequalities in Equation 6 relax the 0–1 values of the indicator variables to any fractional value between 0 and 1.

In implementation, the graph *G*_Δ _is considered implicitly via intersecting intervals. The interval clique inequalities can be generated via a *sweepline *approach (see Figure 6c). The running time depends on the number of iterations needed to solve the LP formulations. Let LP(*n, m*) denote the time taken to solve a linear programming problem on *n *variables and *m *inequalities. Then the worst case running time of the above algorithm is *O*(|Λ|·LP(3|Λ|, 5|Λ| + |Λ_*a*_| + |Λ_*b*_|)). However, the worst-case time complexity happens under the excessive pessimistic assumption that each iteration removes exactly one vertex of *G*_Λ_, namely *χ*_*min *_only, from consideration, which is unlikely to occur in practice as our computational results show. A theoretical pessimistic estimate of the performance ratio of our algorithm can be obtained as follows. Let *α *be the maximum of all the αχmin
 MathType@MTEF@5@5@+=feaafiart1ev1aaatCvAUfKttLearuWrP9MDH5MBPbIqV92AaeXatLxBI9gBaebbnrfifHhDYfgasaacH8akY=wiFfYdH8Gipec8Eeeu0xXdbba9frFj0=OqFfea0dXdd9vqai=hGuQ8kuc9pgc9s8qqaq=dirpe0xb9q8qiLsFr0=vr0=vr0dc8meaabaqaciaacaGaaeqabaqabeGadaaakeaaiiGacqWFXoqydaWgaaWcbaGae83Xdm2aaSbaaWqaaiabd2gaTjabdMgaPjabd6gaUbqabaaaleqaaaaa@348B@ 's over all iterations. Proofs in [32] translate to the fact that the algorithm returns a solution whose total similarity is *at least *1α
 MathType@MTEF@5@5@+=feaafiart1ev1aaatCvAUfKttLearuWrP9MDH5MBPbIqV92AaeXatLxBI9gBaebbnrfifHhDYfgasaacH8akY=wiFfYdH8Gipec8Eeeu0xXdbba9frFj0=OqFfea0dXdd9vqai=hGuQ8kuc9pgc9s8qqaq=dirpe0xb9q8qiLsFr0=vr0=vr0dc8meaabaqaciaacaGaaeqabaqabeGadaaakeaadaWcaaqaaiabigdaXaqaaGGaciab=f7aHbaaaaa@2F52@ times that of the optimum and, if Step 5(b) is omitted from the algorithm, then *α *≤ 4. The value of *α *even with Step 5(b) is much smaller than 4 in practice (*e.g*. *α *= 2.89).

### Simple example

We present a simplified example to illustrate the first iteration of our algorithmic approach for two protein structures *S*_*a *_and *S*_*b *_(Figure 6a) selected for alignment. Here *S*_*b *_is the structure to be aligned to the reference structure *S*_*a*_. We systematically cut *S*_*b *_into fragments of length 4–7 and exhaustively compute a similarity score of each fragment from *S*_*b *_to all possible fragments of equal length in *S*_*a*_. Each fragment pair can be thought of as a vertex in a graph (Figure 6b). *Abusing notations slightly for ease of understanding*, let the vertices be denoted by vertex corresponds to a rectangle in Figure 6c. Suppose we have the following similarity scores for aligned substructures: *σ *(*χ*_1_) = 8, *σ *(*χ*_2_) = 5, *σ*(*χ*_3_) = 7, *σ *(*χ*_4_) = 3 and *σ*(*χ*_5_) = 6. Then, our objective function is to maximize 8xχ1+5xχ2+7xχ3+3xχ4+6xχ5
 MathType@MTEF@5@5@+=feaafiart1ev1aaatCvAUfKttLearuWrP9MDH5MBPbIqV92AaeXatLxBI9gBaebbnrfifHhDYfgasaacH8akY=wiFfYdH8Gipec8Eeeu0xXdbba9frFj0=OqFfea0dXdd9vqai=hGuQ8kuc9pgc9s8qqaq=dirpe0xb9q8qiLsFr0=vr0=vr0dc8meaabaqaciaacaGaaeqabaqabeGadaaakeaacqaI4aaocqWG4baEdaWgaaWcbaacciGae83Xdm2aaSbaaWqaaiabigdaXaqabaaaleqaaOGaey4kaSIaeGynauJaemiEaG3aaSbaaSqaaiab=D8aJnaaBaaameaacqaIYaGmaeqaaaWcbeaakiabgUcaRiabiEda3iabdIha4naaBaaaleaacqWFhpWydaWgaaadbaGaeG4mamdabeaaaSqabaGccqGHRaWkcqaIZaWmcqWG4baEdaWgaaWcbaGae83Xdm2aaSbaaWqaaiabisda0aqabaaaleqaaOGaey4kaSIaeGOnayJaemiEaG3aaSbaaSqaaiab=D8aJnaaBaaameaacqaI1aqnaeqaaaWcbeaaaaa@4BD7@. Figure 6b shows the conflict graph for the set of fragments. A sweep line (shown as dashed lines in Figure 6c) is implicitly constructed (*O *(*n*) time after sorting) to determine which vertices of fragment pairs overlap. A conflict is shown in Figure 6b as edges between vertices. *χ*_1 _and *χ*_5 _do not conflict with any other substructure pairs, while *χ*_2 _and *χ*_4 _conflict with *χ*_3_. For this graph, the constraints in the linear programming formulation are shown in Table 4. The linear programming problem is solved using the BPMPD package [35].

### Similarity Score *σ*

The similarity score *σ*(*χ*_*i, j, k*_) between two aligned substructures λi,ka
 MathType@MTEF@5@5@+=feaafiart1ev1aaatCvAUfKttLearuWrP9MDH5MBPbIqV92AaeXatLxBI9gBaebbnrfifHhDYfgasaacH8akY=wiFfYdH8Gipec8Eeeu0xXdbba9frFj0=OqFfea0dXdd9vqai=hGuQ8kuc9pgc9s8qqaq=dirpe0xb9q8qiLsFr0=vr0=vr0dc8meaabaqaciaacaGaaeqabaqabeGadaaakeaaiiGacqWF7oaBdaqhaaWcbaGaemyAaKMaeiilaWIaem4AaSgabaGaemyyaegaaaaa@3379@ and λj,kb
 MathType@MTEF@5@5@+=feaafiart1ev1aaatCvAUfKttLearuWrP9MDH5MBPbIqV92AaeXatLxBI9gBaebbnrfifHhDYfgasaacH8akY=wiFfYdH8Gipec8Eeeu0xXdbba9frFj0=OqFfea0dXdd9vqai=hGuQ8kuc9pgc9s8qqaq=dirpe0xb9q8qiLsFr0=vr0=vr0dc8meaabaqaciaacaGaaeqabaqabeGadaaakeaaiiGacqWF7oaBdaqhaaWcbaGaemOAaOMaeiilaWIaem4AaSgabaGaemOyaigaaaaa@337D@ is a weighted sum of a shape similarity measure derived from the *cRMSD *value, which is then modified for the secondary structure content of the aligned substructure pairs, and a sequence composition score (*SCS*). Here cRMSD values are the *coordinate root mean square distance*, which are the square root of the mean of squares of Euclidean distances of coordinates of corresponding *C*_*α *_atoms. Formally, for two sets of *n *points **v **and **w**, the cRMSD is defined as ∑1n‖vi−wi‖2
 MathType@MTEF@5@5@+=feaafiart1ev1aaatCvAUfKttLearuWrP9MDH5MBPbIqV92AaeXatLxBI9gBaebbnrfifHhDYfgasaacH8akY=wiFfYdH8Gipec8Eeeu0xXdbba9frFj0=OqFfea0dXdd9vqai=hGuQ8kuc9pgc9s8qqaq=dirpe0xb9q8qiLsFr0=vr0=vr0dc8meaabaqaciaacaGaaeqabaqabeGadaaakeaadaGcaaqaamaaqadabaWaauWaaeaacqWG2bGDdaWgaaWcbaGaemyAaKgabeaakiabgkHiTiabdEha3naaBaaaleaacqWGPbqAaeqaaaGccaGLjWUaayPcSdWaaWbaaSqabeaacqaIYaGmaaaabaGaeGymaedabaGaemOBa4ganiabggHiLdaaleqaaaaa@3C54@.

### cRMSD scaling by secondary structure content

**Figure 7:**
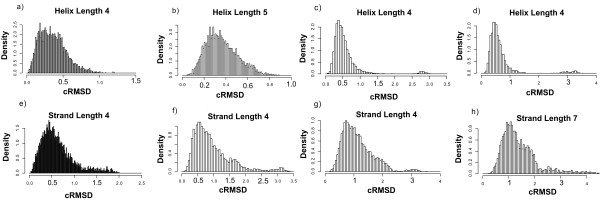
**Secondary Structure cRMSD distributions**. The cRMSD distributions of a) helices of length 4 b) helices of length 5 c) helices of length 6 d) helices of length 7 e) strands of length 4 f) strands of length 5 g) strands of length 6 and h) strands of length 7.

We scale the *cRMSD *according to the secondary structure composition of the two substructures (*λ*^*a *^and λ^*b*^) that compose the substructure pair *χ*. We extracted 1,000 α-helices of length 4–7 (250 of each length) at random from protein structures contained in PDBSELECT 25% [19]. We exhaustively aligned helices of equal length and obtained the *cRMSD *distributions shown in Figure 7(a–d). We then exhaustively aligned equal length β-strands (length 4–7) from a set of 1,000 (250 of each length) strands randomly extracted from protein structures in PDBSELECT 25% [19] and obtained the distributions shown in Figure 7(e–h). For each length, the mean *cRMSD *value of the strands is approximately two times larger than the mean RMSD of the helices. Therefore, we introduce the following empirical scaling factor

s(λa,λb)=∑i=1Nδ(Aa,i,Ab,i)N
 MathType@MTEF@5@5@+=feaafiart1ev1aaatCvAUfKttLearuWrP9MDH5MBPbIqV92AaeXatLxBI9gBaebbnrfifHhDYfgasaacH8akY=wiFfYdH8Gipec8Eeeu0xXdbba9frFj0=OqFfea0dXdd9vqai=hGuQ8kuc9pgc9s8qqaq=dirpe0xb9q8qiLsFr0=vr0=vr0dc8meaabaqaciaacaGaaeqabaqabeGadaaakeaacqWGZbWCcqGGOaakiiGacqWF7oaBdaWgaaWcbaGaemyyaegabeaakiabcYcaSiab=T7aSnaaBaaaleaacqWGIbGyaeqaaOGaeiykaKIaeyypa0ZaaSaaaeaadaaeWaqaaiab=r7aKjabcIcaOiabdgeabnaaBaaaleaacqWGHbqycqGGSaalcqWGPbqAaeqaaOGaeiilaWIaemyqae0aaSbaaSqaaiabdkgaIjabcYcaSiabdMgaPbqabaGccqGGPaqkaSqaaiabdMgaPjabg2da9iabigdaXaqaaiabd6eaobqdcqGHris5aaGcbaGaemOta4eaaaaa@4D9A@

, where

σ(Aa,i,Ab,i)={2,if residues Aa,i and Ab,i are both helix,1,otherwise,
 MathType@MTEF@5@5@+=feaafiart1ev1aaatCvAUfKttLearuWrP9MDH5MBPbIqV92AaeXatLxBI9gBaebbnrfifHhDYfgasaacH8akY=wiFfYdH8Gipec8Eeeu0xXdbba9frFj0=OqFfea0dXdd9vqai=hGuQ8kuc9pgc9s8qqaq=dirpe0xb9q8qiLsFr0=vr0=vr0dc8meaabaqaciaacaGaaeqabaqabeGadaaakeaaiiGacqWFdpWCcqGGOaakcqWGbbqqdaWgaaWcbaGaemyyaeMaeiilaWIaemyAaKgabeaakiabcYcaSiabdgeabnaaBaaaleaacqWGIbGycqGGSaalcqWGPbqAaeqaaOGaeiykaKIaeyypa0ZaaiqabeaafaqaaeGacaaabaGaeGOmaiJaeiilaWcabaGaeeyAaKMaeeOzayMaeeiiaaIaeeOCaiNaeeyzauMaee4CamNaeeyAaKMaeeizaqMaeeyDauNaeeyzauMaee4CamNaeeiiaaIaemyqae0aaSbaaSqaaiabdggaHjabcYcaSiabdMgaPbqabaGccqqGGaaicqqGHbqycqqGUbGBcqqGKbazcqqGGaaicqWGbbqqdaWgaaWcbaGaemOyaiMaeiilaWIaemyAaKgabeaakiabbccaGiabbggaHjabbkhaYjabbwgaLjabbccaGiabbkgaIjabb+gaVjabbsha0jabbIgaOjabbccaGiabbIgaOjabbwgaLjabbYgaSjabbMgaPjabbIha4jabcYcaSaqaaiabigdaXiabcYcaSaqaaiabb+gaVjabbsha0jabbIgaOjabbwgaLjabbkhaYjabbEha3jabbMgaPjabbohaZjabbwgaLjabcYcaSaaaaiaawUhaaaaa@7F97@

to modify the *cRMSD *of the aligned substructure pairs to remove bias due to different secondary structure content. We use DSSP [36] to assign secondary structure to the residues of each protein.

### Sequence composition

The score for sequence composition *SCS *is defined as:

SCS=∑i=1kB(Aa,i,Ab,i),
 MathType@MTEF@5@5@+=feaafiart1ev1aaatCvAUfKttLearuWrP9MDH5MBPbIqV92AaeXatLxBI9gBaebbnrfifHhDYfgasaacH8akY=wiFfYdH8Gipec8Eeeu0xXdbba9frFj0=OqFfea0dXdd9vqai=hGuQ8kuc9pgc9s8qqaq=dirpe0xb9q8qiLsFr0=vr0=vr0dc8meaabaqaciaacaGaaeqabaqabeGadaaakeaacqWGtbWucqWGdbWqcqWGtbWucqGH9aqpdaaeWbqaaiabdkeacjabcIcaOiabdgeabnaaBaaaleaacqWGHbqycqGGSaalcqWGPbqAaeqaaOGaeiilaWIaemyqae0aaSbaaSqaaiabdkgaIjabcYcaSiabdMgaPbqabaGccqGGPaqkaSqaaiabdMgaPjabg2da9iabigdaXaqaaiabdUgaRbqdcqGHris5aOGaeiilaWcaaa@462A@

where *A*_*a, i *_and *A*_*b, i *_are the amino acid residue types at aligned position *i*. *B*(*A*_*a, i*_, *A*_*b, i*_) is the similarity score between *A*_*a, i *_and *A*_*b, i *_based on a modified BLOSUM50 matrix, in which a constant is added to all entries such that the smallest entry is 1.0.

### Combined similarity score

The combined similarity score *σ *(*χ*) of two aligned substructures is calculated as follows:

σ(χi,j,k)=α[C−s(λa,λb)⋅cRMSDk2]+SCS,     (7)
 MathType@MTEF@5@5@+=feaafiart1ev1aaatCvAUfKttLearuWrP9MDH5MBPbIqV92AaeXatLxBI9gBaebbnrfifHhDYfgasaacH8akY=wiFfYdH8Gipec8Eeeu0xXdbba9frFj0=OqFfea0dXdd9vqai=hGuQ8kuc9pgc9s8qqaq=dirpe0xb9q8qiLsFr0=vr0=vr0dc8meaabaqaciaacaGaaeqabaqabeGadaaakeaaiiGacqWFdpWCcqGGOaakcqWFhpWydaWgaaWcbaGaemyAaKMaeiilaWIaemOAaOMaeiilaWIaem4AaSgabeaakiabcMcaPiabg2da9iab=f7aHjabcUfaBjabdoeadjabgkHiTiabdohaZjabcIcaOiab=T7aSnaaBaaaleaacqWGHbqyaeqaaOGaeiilaWIae83UdW2aaSbaaSqaaiabdkgaIbqabaGccqGGPaqkcqGHflY1daWcaaqaaiabdogaJjabdkfasjabd2eanjabdofatjabdseaebqaaiabdUgaRnaaCaaaleqabaGaeGOmaidaaaaakiabc2faDjabgUcaRiabdofatjabdoeadjabdofatjabcYcaSaaa@5956@

In current implementation, the values of *α *and *C *are empirically set to 100 and 2, respectively.

### Similarity score for aligned molecules

The output of the above algorithm is a set of aligned substructure pairs *X *= {*χ*_1_, *χ*_2_, ... *χ*_*m*_} that maximize Equation (1).

The alignment *X *of two structures is scored following Equation (7) by treating *X *as a single discontinuous fragment pair:

σ(X)=α[C−s(X)⋅cRMSDNX2]+SCS.     (8)
 MathType@MTEF@5@5@+=feaafiart1ev1aaatCvAUfKttLearuWrP9MDH5MBPbIqV92AaeXatLxBI9gBaebbnrfifHhDYfgasaacH8akY=wiFfYdH8Gipec8Eeeu0xXdbba9frFj0=OqFfea0dXdd9vqai=hGuQ8kuc9pgc9s8qqaq=dirpe0xb9q8qiLsFr0=vr0=vr0dc8meaabaqaciaacaGaaeqabaqabeGadaaakeaaiiGacqWFdpWCcqGGOaakcqWGybawcqGGPaqkcqGH9aqpcqWFXoqycqGGBbWwcqWGdbWqcqGHsislcqWGZbWCcqGGOaakcqWGybawcqGGPaqkcqGHflY1daWcaaqaaiabdogaJjabdkfasjabd2eanjabdofatjabdseaebqaaiabd6eaonaaDaaaleaacqWGybawaeaacqaIYaGmaaaaaOGaeiyxa0Laey4kaSIaem4uamLaem4qamKaem4uamLaeiOla4caaa@4DCA@

In this case *k *= *N*_*X*_, where *N*_*X *_is the total number of aligned residues.

**Figure 8:**
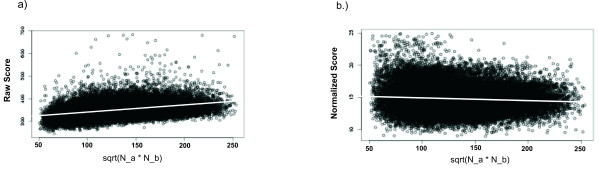
**Similarity Score versus length**. a) Linear fit between *raw similarity score σ *(*X*) (equation 8) as a function of the geometric mean Na⋅Nb
 MathType@MTEF@5@5@+=feaafiart1ev1aaatCvAUfKttLearuWrP9MDH5MBPbIqV92AaeXatLxBI9gBaebbnrfifHhDYfgasaacH8akY=wiFfYdH8Gipec8Eeeu0xXdbba9frFj0=OqFfea0dXdd9vqai=hGuQ8kuc9pgc9s8qqaq=dirpe0xb9q8qiLsFr0=vr0=vr0dc8meaabaqaciaacaGaaeqabaqabeGadaaakeaadaGcaaqaaiabd6eaonaaBaaaleaacqWGHbqyaeqaaOGaeyyXICTaemOta40aaSbaaSqaaiabdkgaIbqabaaabeaaaaa@344A@ of the length of the two aligned proteins (*N*_*a *_and *N*_*b *_are the number of residues in the two protein structures *S*_*a *_and *S*_*b*_). The linear regression line (grey line) has a slope of 0.314. b) Linear fit of the normalized similarity score σ˜
 MathType@MTEF@5@5@+=feaafiart1ev1aaatCvAUfKttLearuWrP9MDH5MBPbIqV92AaeXatLxBI9gBaebbnrfifHhDYfgasaacH8akY=wiFfYdH8Gipec8Eeeu0xXdbba9frFj0=OqFfea0dXdd9vqai=hGuQ8kuc9pgc9s8qqaq=dirpe0xb9q8qiLsFr0=vr0=vr0dc8meaabaqaciaacaGaaeqabaqabeGadaaakeaaiiGacuWFdpWCgaacaaaa@2E85@ (*X*) (equation 9) as a function of the geometric mean of the length of the two aligned proteins. The linear regression line (grey line) has a slope of -0.0004.

**Figure 9:**
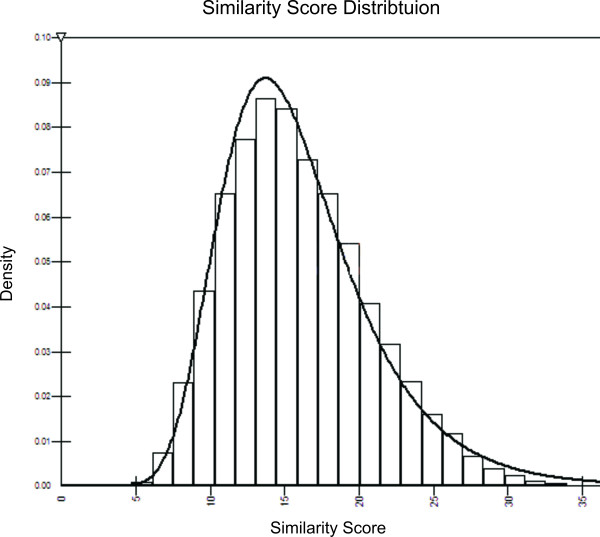
**Similarity Score Distribution**. The distribution of the normalized similarity scores obtained by aligning 200,000 pairs of proteins randomly selected from PDBSELECT 25% [19]. The distribution can be fit to an Extreme Value Distribution, with parameters *α *= 14.98 and *β *= 3.89.

To investigate the effect that the size of each the proteins being aligned has on our similarity score, we randomly aligned 200,000 protein pairs from PDBSELECT 25% [19]. Figure 8a shows the similarity scores *σ *(*X*) (equation 8) as a function of the geometric mean of two aligned structure lengths Na⋅Nb
 MathType@MTEF@5@5@+=feaafiart1ev1aaatCvAUfKttLearuWrP9MDH5MBPbIqV92AaeXatLxBI9gBaebbnrfifHhDYfgasaacH8akY=wiFfYdH8Gipec8Eeeu0xXdbba9frFj0=OqFfea0dXdd9vqai=hGuQ8kuc9pgc9s8qqaq=dirpe0xb9q8qiLsFr0=vr0=vr0dc8meaabaqaciaacaGaaeqabaqabeGadaaakeaadaGcaaqaaiabd6eaonaaBaaaleaacqWGHbqyaeqaaOGaeyyXICTaemOta40aaSbaaSqaaiabdkgaIbqabaaabeaaaaa@344A@. Where *N*_*a *_and *N*_*b *_are the number of residues in *S*_*a *_and *S*_*b*_, respectively. The regression line (grey line) has a slope of 0.314, indicating that *σ *(*X*) is not ideal for determining the significance of the alignment because larger proteins produce higher similarity scores. This is corrected by a simple normalization scheme:

σ˜(X)=σ(X)NX,     (9)
 MathType@MTEF@5@5@+=feaafiart1ev1aaatCvAUfKttLearuWrP9MDH5MBPbIqV92AaeXatLxBI9gBaebbnrfifHhDYfgasaacH8akY=wiFfYdH8Gipec8Eeeu0xXdbba9frFj0=OqFfea0dXdd9vqai=hGuQ8kuc9pgc9s8qqaq=dirpe0xb9q8qiLsFr0=vr0=vr0dc8meaabaqaciaacaGaaeqabaqabeGadaaakeaaiiGacuWFdpWCgaacaiabcIcaOiabdIfayjabcMcaPiabg2da9maalaaabaGae83WdmNaeiikaGIaemiwaGLaeiykaKcabaGaemOta40aaSbaaSqaaiabdIfaybqabaaaaOGaeiilaWcaaa@3AA3@

where *N *is the number of equivalent residues in the alignment is used. Figure 8b shows the normalized similarity score as a function of the geometric mean of the aligned protein lengths. The regression line (grey line) has a negligible slope of -4.0 × 10^-4^. In addition, the distribution of the normalized score σ˜
 MathType@MTEF@5@5@+=feaafiart1ev1aaatCvAUfKttLearuWrP9MDH5MBPbIqV92AaeXatLxBI9gBaebbnrfifHhDYfgasaacH8akY=wiFfYdH8Gipec8Eeeu0xXdbba9frFj0=OqFfea0dXdd9vqai=hGuQ8kuc9pgc9s8qqaq=dirpe0xb9q8qiLsFr0=vr0=vr0dc8meaabaqaciaacaGaaeqabaqabeGadaaakeaaiiGacuWFdpWCgaacaaaa@2E85@ (*X*) can be approximated by an extreme value distribution (EVD) (Figure 9). This allows us to compute the statistical significance given the score of an alignment [37,38].

## Authors' contributions

All authors contributed equally to this paper. All authors read and approved the final manuscript.
